# Investigation of Epistemic Equity in Urban Green Space and Mental Health Research: A Systematic Review

**DOI:** 10.3390/ijerph23020218

**Published:** 2026-02-09

**Authors:** Qin Huang, Kun Liu, Fupeng Li, Yongming Huang, Yanggang Huang, Ryosuke Shimoda

**Affiliations:** 1 Graduate School of Horticulture, Chiba University, 648, Matsudo, Matsudo-shi 271-8510, Chiba, Japan; 23hd0503@student.gs.chiba-u.jp (Q.H.); 25hd0402@student.gs.chiba-u.jp (Y.H.); 2 College of Arts, Shandong Agricultural University, No. 61 Daizong Street, Tai’an 271018, China; kkunlliu@sdau.edu.cn; 3 Faculty of Science and Technology, Keio University, 3-14-1 Hiyoshi, Kohoku-ku, Yokohama 223-8522, Kanagawa, Japan; lifupeng@keio.jp; 4 Faculty of Engineering, Chiba University, 1-33 Yayoi-cho, Inage-ku, Chiba 263-8522, Chiba, Japan; 21wd8307@student.gs.chiba-u.jp

**Keywords:** urban green spaces, mental health, systematic review, epistemic equity, vulnerable populations, cross-sectional design, self-report

## Abstract

**Highlights:**

**Public health relevance—How does this work relate to a public health issue?**

- Identifying who is represented and who is overlooked in green-space and mental-health research is essential for addressing inequities in mental health outcomes.
- Current evidence is shaped by feasibility-driven research practices that systematically exclude vulnerable groups, producing a structurally biased knowledge base.

**Public health significance—Why is this work of significance to public health?**

- This review quantitatively demonstrates the over-representation of convenience samples (healthy adults, university students) and the under-representation of clinical and age-vulnerable populations.
- Evidence is structurally concentrated in “cross-sectional × self-report × healthy adults” designs, limiting generalizability, inclusiveness, and clinical relevance of mental-health findings.

**Public health implications—What are the key implications or messages for practitioners, policy makers and/or researchers in public health?**

- Reliance on feasibility-driven evidence risks perpetuating or worsening mental-health disparities through inequitable green-space policies.
- Equity oriented research, including clinical, age vulnerable and other underserved groups, with greater use of longitudinal and experimental designs, is needed to support inclusive and evidence-based public health and urban planning interventions.

**Abstract:**

Urban public green spaces are widely recognized for having positive effects on mental health, yet existing research shows imbalances in subjects and methodologies. Most studies examine healthy adults and self-reported indicators, giving limited attention to vulnerable populations; this may have led to a gap in evidence regarding “who is represented and who is overlooked.” This study systematically reviewed 235 empirical papers published in 2004–2024 following PRISMA 2020 to examine epistemic equity. The Equity Bias Framework was applied to operationalize epistemic equity by assessing imbalances in study design, psychometric measures, and population sampling. Results showed that the cross-sectional design, self-report, and community-dwelling adults combination accounted for the largest share (*n* = 99, 27.8%), whereas only 9 combinations in total (2.5%) involved clinical populations. The experimental design × self-report × university student patterns (*n* = 14, 3.9%) outnumber all experimental studies involving age-disadvantaged or clinical groups (*n* = 4, 1.1%). These patterns indicate that existing research evidence is shaped more by feasibility and accessibility than by representativeness and clinical relevance, raising concerns about epistemic equity. By introducing the Equity Bias Framework, this study provides a lens on current evidence and direction for research and policy promoting methodological diversity and sample inclusivity.

## 1. Introduction

Rapid urbanization is intensifying mental health challenges worldwide [1,2]. Green spaces, as a non-pharmaceutical and sustainable intervention, are widely recognized for promoting mental well-being [3], demonstrating consistent positive effects in alleviating depression, anxiety, and loneliness while enhancing subjective well-being and emotional regulation [4,5,6]. Beyond mental health, urban green spaces also improve overall quality of life by providing accessible settings for physical activity, informal social interaction, and community engagement. These settings are important for building resilience and promoting everyday well-being [7,8]. During the pandemic, when indoor activities and social venues were restricted, nearby parks and residential green spaces became essential places for safe outdoor activity, psychological comfort, and maintaining social connections [9,10].

However, the impact of green spaces on mental health is not uniform, exhibiting heterogeneity across different demographic groups such as age and health status [5,11,12]. Research further indicates that mental health among vulnerable groups, such as those with psychological disorders, is often more strongly associated with green spaces [13]. Green spaces possess public welfare attributes. As a result, whether their mental health benefits can equitably reach vulnerable groups, such as the elderly, children, and individuals with mental disorders, is increasingly becoming a focal point in urban planning and public health [14,15].

In response, equity-oriented approaches have increasingly integrated health and planning perspectives. From a planning perspective, spatial analysis has been used to evaluate park distribution. It has also informed optimization strategies for site selection that consider spatial and social equity. This improves accessibility for underserved populations [16]. In parallel, a conceptual framework has been proposed that links social cohesion and green gentrification to public health outcomes in historically excluded communities [17]. At the same time, emerging research on “green gentrification” has shown that large-scale urban greening investments can unintentionally raise property values and living costs; this can displace low-income and marginalized residents, thereby limiting who is able to benefit from new green amenities [18,19]. These debates highlight that the social distribution of green space benefits cannot be assumed to be equitable, even when environmental indicators improve. Evidence from mental health research influences both theoretical development and policy formulation. Therefore, assessing whether systematic biases exist across different study designs and sample structures is crucial for evaluating the fairness and representativeness of evidence. Existing studies suggest that vulnerable groups are often “invisible” in policy planning and academic research, leading to a systematic underestimation of their circumstances and needs within the evidence-to-policy chain [20]. For instance, studies indicate that certain methodological combinations (e.g., geographic information systems and questionnaire surveys) are widely adopted [21]. Meanwhile, most research employs cross-sectional designs, potentially introducing regional and national imbalances [22]. This also reflects possible limitations in research subjects and geographic distribution.

In this review, we use the concept of epistemic equity to explore these issues. Based on the concepts of testimonial injustice [23] (e.g., excluding marginalized groups, such as clinical patients, constitutes testimonial injustice and devalues their lived experiences) and epistemic oppression [24] (e.g., a single questionnaire survey becoming the mainstream assessment approach creates an oppressive structure that prevents knowledge about “clinical physiological mechanisms” from being produced and thereby structurally limits the epistemic domain), we define epistemic equity as the fair and inclusive representation of diverse population experiences and assessment methods when constructing research evidence on the impact of urban green spaces on mental health.

Furthermore, the present study distinguishes this concept from three related concepts: health equity, distributional environmental justice, and conventional “research gaps.” While health equity aims to achieve an equitable distribution of health outcomes [25], epistemic equity focuses on the process of producing evidence. For example, if the research process lacks attention to vulnerable populations or appropriate research methods, the necessary evidence to address health inequalities cannot be established. Thus, epistemic equity is a prerequisite for reaching health equity. Distributive environmental justice typically focuses on the distribution of green space resources, such as spatial accessibility to green spaces [26]. However, epistemic equity examines the validity of research evidence. It questions whether research evidence systems primarily derived from healthy individuals are applicable to clinical populations that may require different types of green space interventions. Finally, conventional “research gaps” usually refer to identifying data deficiencies within existing frameworks [27]. However, the epistemic equity perspective not only identifies understudied groups but also focuses on “how knowledge is constructed.”

This review examines how research practices systematically shape which populations and contexts are consistently represented in, or omitted from, the evidence base. Specifically, it operationalizes epistemic equity through the Equity Bias Framework, which examines knowledge production across three dimensions: study design, psychometric measures, and population sampling. Within this framework, we distinguish descriptive imbalances from systemic bias. Imbalance refers to non-structural variations in the frequency of design–measurement–population combinations, which may arise from emergent topics or small literatures. By contrast, bias is conceptualized as a stable, structured preference driven by feasibility considerations (e.g., reliance on cross-sectional designs, self-report instruments, and convenience samples), manifesting as recurrent patterns of specific study design, psychometric measures, and population sampling.

In recent years, researchers have conducted numerous reviews on various topics, including the relationship between green space characteristics and mental health [28], mediating mechanisms (such as social cohesion, physical activity, and environmental stress reduction) [29], systematic reviews on the psychological and behavioral effects of pollen exposure [30], and the relationship between physical activity and mental health [31]. Other reviews have focused on specific populations, such as older adults [32], and university students using campus green spaces [33], whereas bibliometric analyses have revealed emerging research trends and hotspots. However, these reviews primarily focus on the effects and mechanisms of green spaces on psychological outcomes or single-theme analyses of specific populations. They have not systematically compared the evidence across a broad range of studies within a unified framework to address systemic imbalances between different study designs, psychometric measures, and population samples. Crucially, no existing review has yet addressed the following key questions: Which populations are systematically represented in the current research landscape? Which groups remain persistently overlooked? Are these biases fragmented or structural in nature? In other words, there is currently no equity-oriented epistemological framework to quantitatively map biases across study design, measurement, and sampling. Simultaneously, the frequent use of college students as readily accessible samples in mental health research raises concerns about whether this “availability-driven” sampling practice compromises the balance of evidence.

To address this gap, this study systematically reviewed 235 empirical studies on urban green spaces and mental health published between 2004 and 2024, following the PRISMA 2020 statement [34]. We developed an Equity Bias Framework to identify structural imbalances across three dimensions: study design, psychometric measures, and the sample composition. Specifically, this study aims to address three core questions: (1) Do existing studies exhibit systematic preferences in study design and measure selection? (2) Are different social groups fairly represented in research samples, or is there a systematic absence of vulnerable or marginalized populations? (3) How do these biases collectively shape the current evidence structure on urban green spaces and mental health, and how do they impact the representativeness, equity, and policy applicability of the evidence?

## 2. Literature Review

### 2.1. Policies Related to the Mental Health Benefits of Urban Public Green Spaces

With the growing recognition of mental health as a public health priority, increasing attention has been directed toward the psychological benefits of green spaces. This interest has been further amplified by global public health emergencies such as the COVID-19 pandemic.

The WHO introduced the “300-m green space accessibility” guideline, which has been widely integrated into urban public health and planning policies across Europe. Complementarily, the “3-30-300” green space planning rule has gained empirical support, demonstrating that enhanced access to green spaces is associated with improved mental health, decreased reliance on psychotropic medications, and reduced utilization of psychological and psychiatric services [35].

Europe: In the United Kingdom, the National Health Service (NHS) has implemented the Green Social Prescribing initiative, offering a policy-driven framework that connects natural environments with mental health care [36]. Empirical evidence indicates that participation in nature-based activities, such as gardening and nature walks, substantially enhances psychological well-being [37], alleviates symptoms of anxiety and depression [38], and contributes to increased life satisfaction and overall happiness.

America: In the United States, a number of policies and initiatives have emphasized the positive effects of urban green space on mental health, with the Environmental Protection Agency (EPA) stating that green space enhances social connectivity in neighborhoods and reduces the risk of anxiety and depression [39]. The Trust for Public Land has launched the ‘10-Minute Walk’ national initiative, which aims to make high-quality green space within 10 min walking distance of all residents [40], supported by mayors across the United States [41].

Asia: As of 2025, Singapore has established a number of therapeutic gardens, and the National Parks Board aims to establish 30 such gardens by 2030 [42]. Therapeutic Gardens are a core component of the Therapeutic Horticulture Program, which provides horticultural interventions for the aging and mental health risk groups [43]. In China, the “Healthy China Action: Healthy Environment Promotion Action Implementation Program (2025–2030)” mentions the “Healthy and Comfortable Home Environment Promotion Action”, which covers mental health, and the “Friendly Community Environment Construction Action”, which promotes the construction and maintenance of community green spaces and trails, but there is no direct reference to the role of green spaces in mental health [44].

Africa: African Green Recovery Action Plan (2021–2027) refers to the benefits of green and blue infrastructure in providing recreational space [45]. Although recreational space has been shown to be indirectly related to mental health, the plan does not explicitly mention the “mental health effects of green spaces”.

Comparing countries across regions, Europe and the United Kingdom are at the forefront of institutionalization and quantifiable policy targets. The United States focuses on community engagement and accessibility, while Singapore directly embraces green spaces as one of the pathways for mental health interventions. In contrast, China and Africa tend to reflect the mental health value of green spaces more indirectly, often within health or climate adaptation frameworks, and policies explicitly focusing on mental health as a core objective remain relatively limited. This variation reflects differences in policy objectives, implementation approaches, and health prioritization across locations.

### 2.2. Role of Green Spaces in Mental Health

Most studies have concentrated on the impact of green spaces on pathological mental states, including the reduction of anxiety [46], alleviation of depression [47], and mitigation of emotional disorders such as loneliness and stress [48,49]. Additionally, some research has investigated psychological recovery and stress alleviation in response to traumatic experiences [50]. In recent years, there has been a growing emphasis on positive mental health outcomes such as well-being and resilience. These include perceived enhancements in psychological well-being [51,52], subjective experiences of restoration [53], such as attention restoration [54], promotion of mindfulness [55], reductions in mentally unhealthy days [56], improved emotional stability [57], and decreased psychological distress [58].

Some studies have also examined behavioral and physiological dimensions, including improved sleep quality and behavioral functioning [59,60], and regulation of cortisol levels and blood pressure [61]. The broad spectrum of explored mental health outcomes underscores the increasing recognition of the psychological benefits of green spaces and offers compelling evidence of their efficacy. In the post-pandemic context, these benefits have become increasingly varied and salient.

### 2.3. Measurement Techniques of Mental Health Indicators

Regarding the assessment of mental health dimensions, self-reported questionnaires are frequently employed. Standardized psychological instruments include the Multidimensional Mood Questionnaire (MDMQ) [62], validated Positive Psychology Scales [63], Subjective Well-Being (SWB) Scales [64], the SF-12v2 Health Survey [65], and the Brief Symptom Inventory (German version) [66]. Additionally, some studies have utilized self-constructed questionnaires to assess variables such as psychotropic substance use frequency [67] and general well-being [68]. Objective physiological and biochemical indicators, such as heart rate variability (HRV) [69], and electrodermal activity [70,71], are also utilized in some studies as objective measures of mental health.

In recent years, there has been a growing adoption of big data and health databases, incorporating sources such as medical records (e.g., the density of registered hospitalized patients diagnosed with depression) [72], social media content analyses [73], facial expression recognition, and sentiment scoring derived from social media platforms [74]. Moreover, interviews have been predominantly used in studies involving small sample sizes and specific population groups [75]. A wide variety of methods exist for measuring mental health outcomes in public green spaces, and with the advancement in technology, big data approaches utilizing hospital data and social media have begun to emerge. However, there remains a dearth of review-type literature that quantitatively analyzes preferences for measurement methods. In addition, little attention has been paid to the co-occurrence frequency of measurement methods across different mental health categories.

### 2.4. Characteristics and Measurement of Urban Green Spaces Influencing Mental Health

The quantifiable spatial and physical attributes of urban green spaces have garnered significant attention in research investigating their impact on mental health. Commonly utilized metrics include the total area of public green space [76], vegetation cover as measured by the Normalized Difference Vegetation Index (NDVI) [77], green volume or tree density [78], and greenway exposure, all of which are often assessed through GIS-based buffer analyses (e.g., within a 0–5 km street network radius) [79]. Additional indicators comprise green volumetric ratios derived from LiDAR point cloud data and visual characteristics such as color richness [80]. Furthermore, the provision of ecosystem services, such as thermal regulation, air purification, noise attenuation, and plant biodiversity, has been positively associated with psychological well-being [81].

Subjective experiences and sensory stimuli also play a critical role in shaping mental health outcomes. Pertinent perceptual features include perceived naturalness [82], low-level visual elements (e.g., curved edges) [83], seasonal variability [84], and sensory-specific experiences characterized by qualities such as “serenity”, encompassing calmness, an absence of urban noise, and the presence of natural elements like water bodies or birdsong [85]. Functional and usability aspects of green spaces are often evaluated through parameters such as openness, accessibility, and availability of supporting infrastructure, as well as engagement in recreational, social, or physical activities (including walking and sports) [86,87].

Across both spatial and perceptual dimensions, on-site experiences and self-reported surveys remain the predominant methodologies for assessment [88]. In addition, VR-based simulations have gained traction in replicating real-world scenarios and capturing subjective perceptions [89]. Remote sensing and GIS technologies are widely used to quantify spatial and ecological features of green spaces [78]. The integration of remote sensing with on-site evaluations enables a comprehensive measurement of ecological attributes alongside subjective experiences [90]. With the advancement of big data analytics, emerging techniques include the utilization of geotagged social media data and urban street view image recognition to extract green space characteristics [91]. However, the current application of these techniques remains relatively limited.

## 3. Materials and Methods

### 3.1. Implementation of the Equity Bias Framework

To put the concept into practice, we developed the Equity Bias Framework, which examines the research process through three interrelated dimensions: (1) study design, (2) psychometric measures, and (3) population sampling. These dimensions were chosen because they are critical decision points in the research process where systemic exclusion is most likely to occur. Regarding sample composition, overreliance on “WEIRD” populations (Western, Educated, Industrialized, Rich, Democratic) has been shown to undermine external validity and obscure the experiences of marginalized groups [92]. Grounded in the concept of epistemic oppression [24], we also critically examined study design and measurement instruments. The scarcity of shared cognitive resources often leads to the neglect of marginalized groups’ lived realities. For example, when standardized self-report questionnaires that demand high literacy or cognitive function dominate, they structurally exclude specific populations from contributing to evidence systems.

This framework rests on two core assumptions. First, we hypothesize that the feasibility-driven principle (“the path of least resistance”) operates multidimensionally, prompting researchers to prioritize easily accessible populations, low-burden measurement tools, and straightforward study designs. Second, we contend that bias is cumulative and structural. While reliance on a single methodological approach may not be significant in isolation, the co-occurrence of biases across three dimensions (population sampling, study design, and measurement methods) collectively constitutes structural exclusion. This framework is designed to evaluate the representativeness and inclusiveness of the overall research evidence base. The scope of this framework is defined at the aggregate level and designed to assess the epistemic inclusion of the collective evidence base rather than the internal validity of individual studies. Unlike risk-of-bias tools (e.g., Cochrane RoB), which evaluate the internal validity of specific findings, this framework evaluates the representativeness of the overall evidence structure.

### 3.2. Eligibility Criteria and Data Extraction

This review follows a previously proposed methodological framework [93], grounded in the international standards for systematic reviews outlined by the PRISMA 2020 guidelines [34]. This systematic review was conducted in accordance with the PRISMA 2020 guidelines and was not prospectively registered. This approach has been employed in several related studies. The systematic review process comprises six stages: (1) Scoping, (2) Planning, (3) Identification and Search, (4) Screening, (5) Eligibility Assessment, and (6) Presentation and Interpretation of Results. Stages 2 and 4 were conducted by a single researcher, while Stage 5 involved two independent reviewers. In Stage 5, Reviewer 1 evaluated each article, and Reviewer 2 cross-checked the assessments for consistency. Discrepancies were discussed and resolved collaboratively by both reviewers in consultation with the study authors, with final decisions made by consensus. No automation tools were used during the screening or eligibility assessment process, and no formal risk of bias assessment tool was applied. However, methodological rigor was ensured through independent verification and cross-checking between reviewers.

In the scoping phase, the search strategy was structured around three dimensions: Spatial context (e.g., “urban,” “city”), Green space types (e.g., “park,” “green space,” “green infrastructure,” “green corridor*,” “urban forest”), and Mental health (e.g., “mental health,” “depress*,” “anxi*,” “stress,” “psychological,” “subjective wellbeing,” “emotional restoration”). During the planning phase, these keywords were combined using Boolean operators to generate the following search query:

TS = ((“urban” OR “city”) AND (“park*” OR “green space” OR “green infrastructure” OR “green corridor*” OR “urban forest”) AND (“mental health” OR “depres*” OR “anxi*” OR “stress” OR “psychological” OR “subjective wellbeing” OR “emotional restoration” OR “restorative quality” OR “attention” OR “perceived safety” OR “affect*” OR “emotion*” OR “mental” OR “percept*” OR “restorative*”)). The full Web of Science search strategy is reported at PRISMA-S level of detail in Supplementary Text S1, including the exact search string as executed, search fields, search date, language, and document-type limits.

During the identification and search phase, the Web of Science database was selected, covering literature published between January 2004 and December 2024. The database was searched on 25 April 2025 to ensure coverage up to December 2024. An initial pool of 12,524 records was retrieved. In the screening phase, 1860 records that were non-English publications or non-article document types were excluded. An initial filter based on Web of Science subject categories was applied to exclude records from domains that were conceptually unrelated to the study objectives, such as physics, geology, and entomology. To prevent the inadvertent exclusion of relevant interdisciplinary research, we manually verified high-risk categories (e.g., transportation and human geography) and randomly spot-checked other excluded fields. This process confirmed the irrelevance of these records. In total, 7837 unique records were excluded through category-based filtering (see Supplementary Table S1 for full list of excluded Web of Science topic filters and record counts). Additionally, a total of 16 records could not be retrieved due to inaccessible full texts and were therefore excluded from the eligibility assessment stage. In the eligibility assessment phase, 2575 records were manually excluded if they met either of the following criteria:

(i) documents on green spaces that are not comparable or not typically accessible as everyday public environments (e.g., national nature reserves, ecological conservation zones for ecosystem services rather than public use, areas without public functions, or private/gated green spaces such as golf courses or gated residential gardens).
(ii) documents that did not address the psychological or mental health effects of green spaces, or that mentioned such effects only as background context without providing direct empirical verification. For instance, several studies discussing the aesthetic or recreational value of green spaces without providing empirical evidence of psychological or mental health outcomes were excluded at the eligibility assessment stage.

**Figure 1:**
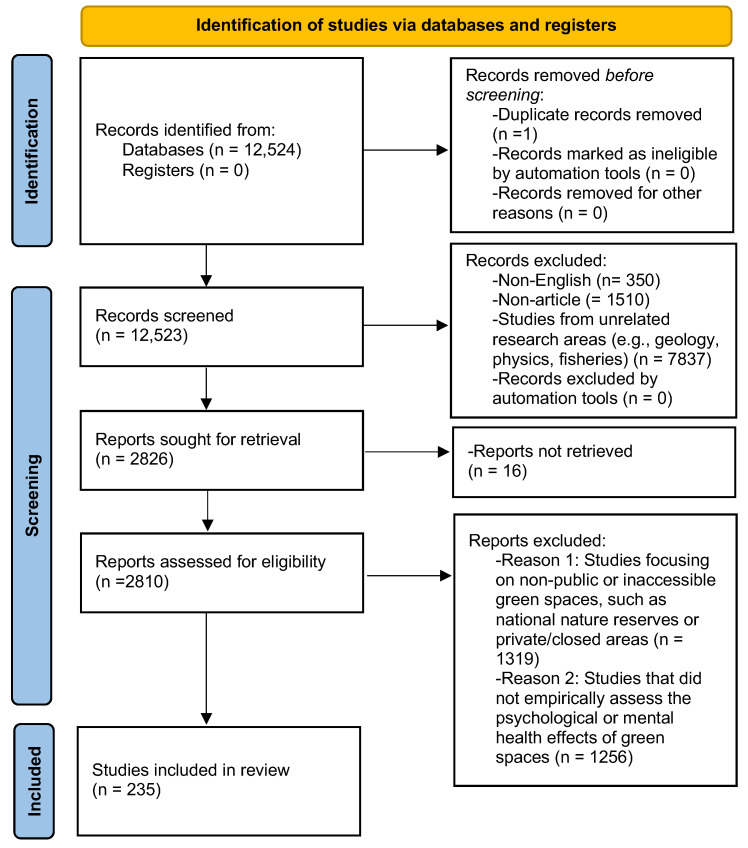
PRISMA flow diagram of the study selection process (modified from [34]).

After this process, 235 studies were included. The workflow is illustrated in Figure 1. In addition to PRISMA 2020, we implemented a multi-stage screening procedure to enhance reliability. Two reviewers first piloted the inclusion and exclusion criteria on a random sample of 80 records and refined the decision rules following discussion. The remaining records were then screened by one reviewer using the final criteria, with brief justifications recorded for each decision (e.g., “Excluded: no mental health outcome”). To mitigate single screener bias, two additional reviewers subsequently audited the screening decisions and resolved any discrepancies through consensus meetings. Full details of the calibration and verification procedures are reported in Supplementary Text S1.

Following full-text review of the 235 included studies, core information was extracted into a standardized Excel spreadsheet, including: year of publication; study location; green space characteristics; mental health dimensions; psychometric measures; study design; and population samples. Initial coding was performed by one researcher and independently verified by another, with discrepancies resolved through consensus. The complete coding scheme, including variable definitions, decision rules, and handling of “Not Reported” (NR) values, is detailed in Supplementary Text S2 (Codebook). To ensure transparency and facilitate future reuse, the extracted dataset was coded according to these definitions. Granularity is provided in Supplementary Table S2. Both Supplementary Text S2 and Supplementary Table S2 are provided as Supplementary Materials alongside the article and can be accessed via the journal’s website.

For data analysis, descriptive statistics (frequencies and percentages) were calculated to summarize these variables, and temporal trends were visualized. Additionally, cross-distributions were examined (e.g., green space characteristics × mental health dimensions; study design × population samples). To visualize methodological pathways linking study designs and measurement approaches, a Sankey diagram was generated using the web-based tool SankeyMATIC (https://sankeymatic.com/, accessed on 11 August 2025). The nodes on the left represent study design categories, and the nodes on the right represent types of mental health measurements. For each included study, we identified the study design and all the types of mental health measurements used. A separate flow was created for each observed design-measurement combination. The width of each flow is proportional to the number of studies employing that specific combination. Since individual studies often employ multiple measurement tools or assess various mental health outcomes, percentages were calculated based on the total frequency of measurement dimension pairings (rather than the number of studies). This approach quantifies methodological preferences across all reported combinations.

### 3.3. Counting Rules and Units of Analysis

Given the methodological heterogeneity of the included literature, individual studies often employed multiple designs, measurement tools, or simultaneously assessed several mental health dimensions. Relying solely on the number of studies (*n* = 235) can obscure important data granularity. To ensure accuracy and analytical depth, we applied different units of analysis for specific summaries, defined as follows:

(1) Single Article (*n* = 235): This unit is applied to general literature characteristics (e.g., publication year, country of origin).
(2) Pairing of green space characteristics and psychological outcome dimensions (*n* = 1193): In the crosstab heatmap, it counts each specific instance of a particular green space characteristic (e.g., NDVI) compared to a particular mental health outcome dimension in the study.
(3) Path of study designs and psychometric measures (*n* = 363): In the Sankey diagram, the unit of analysis represents the flow from study design to psychometric measures. This number exceeds the total number of studies because mixed-method studies contributed multiple different methodological records.
(4) Mental health outcome categories and psychometric measures pairings (*n* = 783): The analysis unit is a specific pairing between the measurement tool and the mental health dimension (e.g., a study that used both self-report scales and Physiological and biochemical indicators contributed two different pairings).
(5) Analysis triads (*n* = 356): For the comprehensive analysis of study patterns, the analysis unit is the “study design × psychometric measures × population sampling” triad.

### 3.4. Categorization of Mental Health Dimensions

In order to systematically conceptualize the pathways through which urban green spaces influence mental health, this study, based on an extensive review of relevant literature, synthesized the Diagnostic Framework for Clinical Psychiatry [94], the WHO’s Mental Health Action Plan [95], and recent practices of categorizing psychological research in urban environments [96]. The mental health effects were subsequently classified into five dimensions. (1) Emotional Disorders, covering common negative emotional symptoms such as depression, anxiety. These represent core indicators in urban mental health research and are commonly measured by standardized scales such as the PHQ-9 and GAD-7. (2) Stress and Trauma, including psychological stress and post-traumatic reactions to life stress or unexpected events (e.g., disasters, epidemics), with assessment tools such as PSS (Perceived Stress Scale) and PTSD Checklist. (3) Well-being and Resilience, referring to the subjective sense of well-being, positive emotions, and psychological resilience in the face of adversity; commonly used scales in this dimension include WHO-5 and WEMWBS. (4) Sleep and Behavioral Functioning, involving sleep quality, biorhythm, self-control and daily behavioral efficiency. (5) Neuropsychological and Physiological, including cognitive function, concentration, and physiological indicators related to psychological states (e.g., heart rate variability, cortisol concentration), reflecting the mechanism of psychological-physiological interaction. This five-dimensional classification provides the basis for the subsequent extraction of literature information.

For each mental health outcome domain, all reported measures were extracted. When multiple instruments or time points were reported within a single study, we prioritized the primary outcome specified by the authors for synthesis. If no primary outcome was specified, all relevant measures were recorded; however, each study was still counted only once per outcome domain in the summary analyses.

### 3.5. Categorization of Urban Green Space Characteristics

To systematically identify the mechanisms through which urban green spaces influence mental health, this study draws upon a comprehensive review of existing literature. It references the three-dimensional model of green infrastructure, comprising physical structure, ecological services, and opportunities for social interaction [97]. The study also incorporates structural and ecological dimensions from health research, as well as spatial mapping frameworks for characterizing urban green spaces [98]. Key green space attributes examined in mental health research include structural indicators (e.g., NDVI) [99], perceived aesthetics, and dimensions of social accessibility and usability [100].

Based on this synthesis, a framework was developed to categorize green space characteristics into four dimensions used in this study:

(1) Physical Structure: Physically measurable features of green spaces such as total area, NDVI (Normalized Difference Vegetation Index), and canopy cover.
(2) Ecological Functions: Regulatory ecosystem services provided by green spaces including temperature regulation, air purification, noise attenuation, and biodiversity support.
(3) Perceived Naturalness and Aesthetics: Subjective perception of naturalness, visual appeal, spatial enclosure, and other experiential qualities.
(4) Social and Recreational Usability: Aspects such as accessibility, openness, availability of supportive infrastructure, and the facilitation of social and recreational activities.

### 3.6. Categorization of Study Populations

In existing reviews on green spaces and mental health, researchers have focused on specific populations. For instance, some reviews emphasize adolescents and children, treating developmental stages as unique vulnerability factors [101,102]. Several additional studies have focused on older adults, revealing age-related limitations in access to green spaces and mental health burdens [103]. Additional studies have focused on clinically or psychosocially burdened groups and other vulnerable populations, explicitly examining whether community green spaces can alleviate psychological distress among high-risk individuals rather than the general adult population [104]. College students, as a conveniently sampled institutional group, have also drawn attention [33]. Such research focuses indicate that population types are implicitly categorized based on vulnerability and sampling convenience. Participants described as “urban residents” or “urban adults” without further demographic specification are classified in this review as community-dwelling adults. This categorization aligns with a prior study on green spaces, which treats such samples as adult populations [105]. In summary, the present review integrates the sampling into four population categories:

(1) institutional convenience populations
(2) community-dwelling adult populations
(3) age-specific vulnerable populations
(4) clinical or high-risk populations

### 3.7. Quality Appraisal and Data Synthesis Strategy

A structured data extraction process was employed to ensure consistency. However, due to the considerable heterogeneity in study designs (ranging from cross-sectional to experimental), diverse conceptualizations of green space exposure, and the wide array of psychometric instruments used across the 235 included studies, statistical or meta-analytic techniques were not applied. Instead, we adopted a descriptive and narrative synthesis approach to summarize the findings qualitatively, focusing on the distribution of evidence rather than aggregating effect sizes.

Regarding quality assessment, a formal risk-of-bias assessment was not performed. This decision was based on two considerations. First, substantial heterogeneity in study designs made the use of a single, standardized quality metric (e.g., MMAT or RoB 2) less applicable. Second, the primary aim of this systematic review was to examine “epistemic equity”, specifically to characterize the volume and structural distribution of research efforts (i.e., who is represented versus who is overlooked), rather than to synthesize clinical efficacy. Reporting completeness (e.g., clarity of population demographics and measurement tools, and whether psychometric reliability indices were reported) was therefore prioritized over an assessment of internal validity. This limitation is acknowledged, and the findings should be interpreted as reflecting the current research landscape and structural methodological patterns rather than confirming causal robustness.

## 4. Results

### 4.1. Structural Evolution of Research Focus: Who and What Are Studied

**Figure 2:**
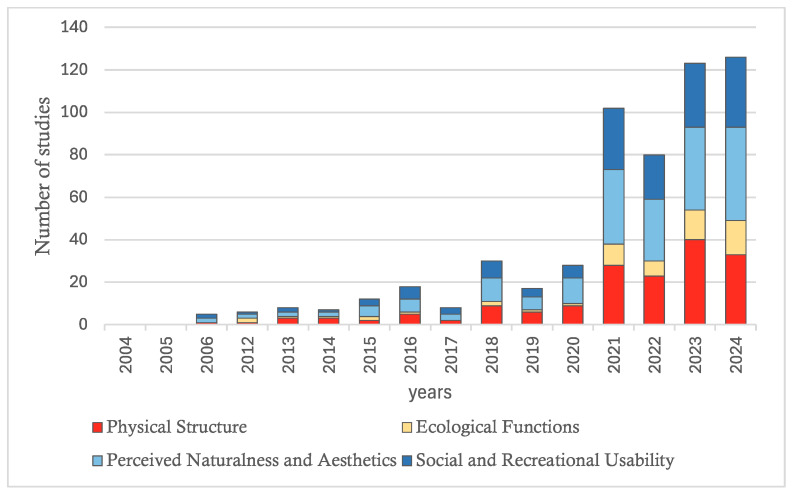
Annual number of studies on the four characters of public green space attributes in urban green space and mental health research (2004–2024): “Physical Structure”, “Ecological Functions”, “Perceived Naturalness and Aesthetics”, and “Social and Recreational Usability”.

Research activity surged significantly following the COVID-19 pandemic (Figure S1). Table S3 reveals a severe geographic imbalance. Studies are concentrated in Asia (55.74%) and Europe (24.26%), while Africa (1.28%) and South America (1.70%) remain underrepresented in the English-language Web of Science dataset. The overall trend indicates a substantial increase in scholarly and public interest in the psychological functions of urban green spaces during the COVID-19 pandemic, which significantly accelerated research activity in this domain. According to Figure 2, there is a marked disparity in the degree of scholarly attention devoted to different dimensions of green space features in studies addressing urban public green spaces and mental health. Among the four dimensions related to public green spaces, “Perceived Naturalness and Aesthetics” accounts for the highest proportion of studies, with a cumulative total of 198 articles, representing 84.26% of all reviewed publications, which substantially exceeds the representation of other dimensions. “Physical Structure” ranks second, with 165 studies, constituting 70.21% of the total. This is followed by “Social and Recreational Usability,” with 149 studies (63.40%). In contrast, “Ecological Functions” is the least represented, comprising only 58 studies (24.68%).

“Perceived Naturalness and Aesthetics” has shown a sustained increase, reaching 11 studies in 2018 and 12 in 2020, and peaking at 44 in 2024, which represents the highest annual count recorded for this dimension. “Physical Structure” has exhibited a clear upward trajectory, with steady growth until 2020, followed by a sharp increase beginning in 2021 and peaking at 40 studies in 2023 and 33 in 2024. “Social and Recreational Usability” displays a consistent upward trend, rising from 6 studies in 2020 to 29 in 2021, and reaching 33 in 2024. In contrast, “Ecological Functions” has generally received limited attention. Although it has experienced gradual growth, increasing from 2 studies in 2012 to a peak of 16 in 2024, it remains comparatively less represented than the other dimensions.

Overall, “Physical Structure” gained traction earlier, while “Perceived Naturalness and Aesthetics” and “Social and Recreational Usability” saw significant growth after 2020. “Ecological Functions,” though increasingly recognized, remains underrepresented in terms of research volume.

**Figure 3:**
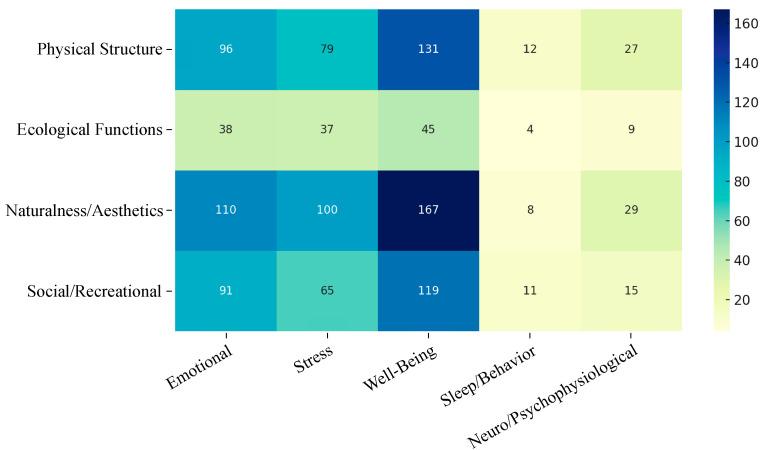
This is a cross-tabulation heatmap showing the relationship between green space characteristics and psychological outcome dimensions. The heatmap illustrates the frequency of reported associations. A total of 1193 associations between characteristics and outcomes were identified across the dataset. Abbreviations: Naturalness/Aesthetics, Perceived Naturalness and Aesthetics; Social/Recreational, Social and Recreational Usability; Emotional, Emotional Disorders; Stress, Stress and Trauma; Well-Being, Well-Being and Resilience; Sleep/Behavior, Sleep and Behavioral Functioning; Neuro/Psychophysiological, Neuropsychological and Physiological.

In terms of outcomes, studies on subjective well-being and resilience dominate the field, whereas objective neuropsychological and physiological indicators have received comparatively limited attention (Figure S2). According to Figure 3, “Perceived Naturalness and Aesthetics × Well-being and Resilience” (*n* = 167) and “Physical Structure × Well-being and Resilience” (*n* = 131) predominate in the literature, indicating a strong focus on subjective perceptions of green spaces and general well-being outcomes. In contrast, “Ecological Functions × Sleep and Behavioral Functioning” (*n* = 4) and “Ecological Functions × Neuropsychological and Physiological” (*n* = 9) are rarely examined, suggesting a disproportionate focus on affective and cognitive outcomes over physiological or behavioral domains.

Overall, these findings indicate that current research evidence systematically focuses on perceptual experiences and positive psychological states, while significant research gaps remain in interdisciplinary areas involving ecological or physiological mechanisms. This pattern reveals an epistemic inequity in how mental health evidence is constructed—subjective, self-report dimensions are legitimized, whereas ecological and physiological pathways remain marginal.

### 4.2. Study Design × Psychometric Measures Pattern: Self-Report Dominates, with Scarce Objective and Behavioral Indicators

**Figure 4:**
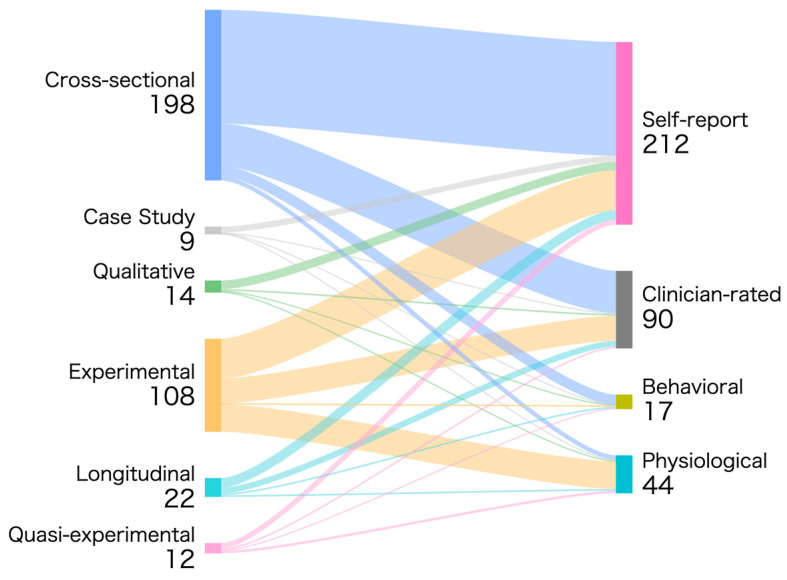
Sankey visualization of methodological pathways linking study designs and psychometric measures. Abbreviations: Cross-sectional, Cross-sectional study; Longitudinal, Longitudinal study; Experimental, Experimental research; Quasi-experimental, Quasi-experimental study; Case study, Case study design; Qualitative descriptive, Qualitative descriptive design; Self-report, Self-report psychometric measures; Clinician-rated, Clinician-rated psychometric measures; Physiological, Physiological and biochemical indicators; Behavioral, Behavioral and observational measures. Colors are used solely for visual distinction and do not encode additional analytical meaning.

The Sankey diagram mapping study design to psychometric measures across 235 studies, totaling 363 records, reveals pronounced methodological co-occurrence patterns (Figure 4). Cross-sectional designs appear most frequently (198 instances, 54.5%) and form the primary pathway leading to self-reported measurements. Experimental studies, as the second-most common design category (108 instances, 29.8%), exhibit a more dispersed measurement pathway, flowing toward self-reports, clinician ratings, and physiological or biochemical indicators. However, self-reports still constitute the broadest connection channel.

Self-report instruments accounted for the highest proportion among all psychometric measures (212 instances, 58.4%), serving as the primary output for cross-sectional studies, qualitative descriptive research, and some experimental studies. Clinician ratings (90 instances, 24.8%) and physiological or biochemical indicators (44 instances, 12.1%) primarily originated from experimental designs and longitudinal studies, indicating their limited overall prevalence but relatively concentrated sources within the evidence base. Behavioral and observational measurements accounted for the lowest proportion of all links (17 instances, 4.7%), primarily originating from cross-sectional studies, with significantly lower connectivity than other pathways. Overall, the flow relationships between study design and measurement methods clearly reveal the strongest coupling feature: “cross-sectional study → self-report measurement.” While objective measurement methods receive inputs from multiple design types, their overall proportion remains significantly lower than subjective self-report indicators.

### 4.3. Feasibility Bias and the Construction of Mental Health Evidence

**Figure 5:**
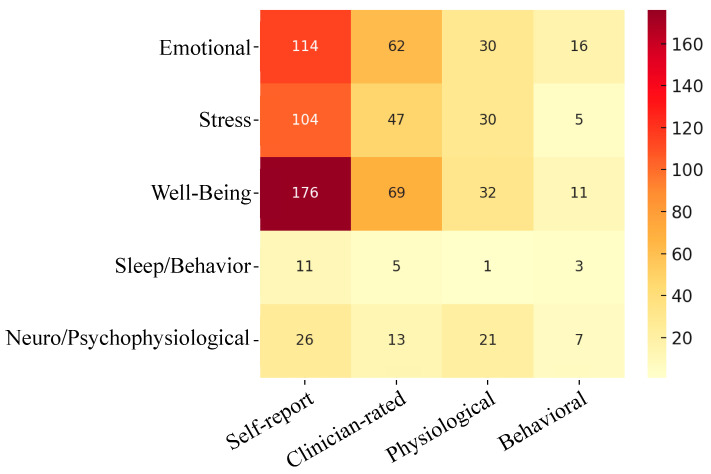
Distribution of mental health outcome categories across measurement. Abbreviations: Emotional, Emotional disorders; Stress, Stress and trauma; Well-Being, Well-Being and resilience; Sleep/Behavior, Sleep and behavioral functioning; Neuro/Psychophysiological, Neuropsychological and physiological; Self-report, Self-report psychometric measures; Clinician-rated, Clinician-rated psychometric measures; Physiological, Physiological and biochemical indicators; Behavioral, Behavioral and observational measures.

Figure 5 displays the distribution of measures and mental health pairings. Among the 235 included studies, a total of 783 pairing combinations were identified. “Self-report measures” dominated the evidence base, accounting for 431 instances (55.0%), followed by “Clinician-rated psychometric measures” (*n* = 196, 25.0%). The use of “Physiological and biochemical indicators” (*n* = 114, 14.6%) and “Behavioral and observational measures” (*n* = 42, 5.4%) was less common.

In these pairings, the combination of “self-report measures” with emotion-related indicators was notably concentrated. Specifically, “self-report measures” of “Well-Being and Resilience” (*n* = 176, 22.5%), “Stress and Trauma” (*n* = 104, 13.3%), and “Emotional Disorders” (*n* = 114, 14.6%), which together constituted the largest share of self-report-based evidence in the dataset. In contrast, objective observational methods were rarely employed to assess these or any other mental health dimensions. For instance, within any single mental health domain, “Physiological and biochemical indicators” appeared no more than 32 instances, with only one instance paired with “Sleep and Behavioral Functioning”. Furthermore, “Behavioral and observational measures” constituted only a small fraction of the total evidence base. Notably, the specific combination of this measure with the “Sleep and Behavioral Functioning” domain occurred in just three instances, representing a mere 0.38% of the total pairings. Similarly, “Clinician-rated psychometric measures” were occasionally used to assess affective structures like “Well-Being and Resilience” (*n* = 69, 8.8%) and “Emotional Disorders” (*n* = 62, 7.9%). No pronounced high-frequency patterns of clinician-administered or objective measures were observed across cognitive, behavioral, or neurophysiological domains, indicating that these areas warrant further investigation. Overall, these data reveal consistent methodological convergence in self-reported emotional structures, reflecting the current evidence base on green spaces and mental health where easily implemented subjective assessments dominate, while objective, behavioral, and clinician-rated measures remain relatively neglected.

### 4.4. Systemic Pattern of Epistemic Inequity

A quantitative synthesis of 235 studies revealed co-occurrence patterns across study designs, psychometric measures, and population distributions (Table 1). This analysis explored how preferences for study design and measures influence evidence in research, and which populations remain underrepresented. Among the 96 possible triad types (study design × population sampling × psychometric measures), a small subset of recurring triads accounted for the majority of observed instances, indicating a highly concentrated methodological preference within the literature. In total, 356 triad instances were identified across the 235 included studies.

The most significant triad combination was Cross-sectional study × Self-report measures × Community-dwelling adult populations, comprising 99 triads (27.8%) and representing the most prevalent combination in the dataset. The next most common triads were: Experimental research × Self-report measures × Community-dwelling adult populations (27 triads; 7.6%) and Cross-sectional study × Self-report measures × Age-specific vulnerable populations (22 triads; 6.2%). Together, these top three combinations accounted for approximately 41.6% of all identified triads.

The combination of Physiological and biochemical indicators or Clinician-rated psychometric measures was notably underrepresented. For example: Experimental research × Physiological and biochemical indicators × Community-dwelling adult populations appeared in 18 triads (5.1%), whereas Experimental research × Clinician-rated psychometric measures × Community-dwelling adult populations appeared in 16 triads (4.5%). Furthermore, regardless of study design or psychometric measures, the total number of triads involving clinical populations was only *n* = 9 (2.5%), indicating that research on clinical groups remains extremely limited across all methodological configurations. Behavioral and observational measures appeared in 17 triads (4.8%), with 11 triads (3.1%) combining adult populations and cross-sectional designs, and only 2 triads (0.6%) involving age-specific vulnerable populations or clinical or high-risk populations. Experimental research × self-report measures × institutional convenience populations constituted a small yet distinct high-frequency pattern, comprising 14 ternary combinations (3.9%). This figure exceeds the total number of combinations including experimental research paired with any specific population (age-specific vulnerable populations and clinical or high-risk populations) (*n* = 4, 1.1%). This reflects a research methodology tendency toward selecting easily accessible yet non-representative samples.

Overall, the frequency matrix reveals a systematic triadic bias: Cross-sectional study × Self-report measures × Community-dwelling adult populations (27.8%) dominate the empirical research landscape, whereas studies employing Experimental research or Longitudinal study × Clinician-rated psychometric measures/Physiological and biochemical indicators/Behavioral and observational measures × Age-specific vulnerable populations or clinical or high-risk populations are virtually absent (<1.5%). This pattern highlights a structural methodological imbalance, suggesting that feasibility and accessibility of research take precedence over representativeness and clinical relevance in the current landscape of green spaces and mental health evidence.”

## 5. Discussion

### 5.1. Impact of the Global Public Health Crisis on Research Motivation

Since the onset of the COVID-19 pandemic, there has been a notable increase in the number of studies on green spaces and mental health. At the same time, there has been a discernible shift in the focus of this research toward specific green space features associated with mental health. In particular, there has been a substantial rise in investigations emphasizing the mental health benefits of urban public green spaces within the categories of “Perceived Naturalness and Aesthetics” and “Social and Recreational Usability” since 2020.

For example, one study reported that during the pandemic, public engagement in social activities within urban parks appeared to be closely linked to subjective judgments of “psychological comfort” [106]. Wang identified green and blue spaces as forms of “psychological self-repair” during the epidemic, noting that this reparative function was associated with individuals’ subjective perceptions of spatial characteristics [107].

Several studies have shown that the richness of natural elements (e.g., species diversity) is significantly associated with subjective psychological benefits [108,109] and that higher quality landscapes are linked to reported stress-reducing and restorative effects [54,110]. Together, these findings suggest a positive relationship between individuals’ subjective perceptions of green spaces and their psychological recovery, particularly in terms of stress and anxiety reduction.

In addition, one study compared subjective accessibility with objective accessibility and found that subjective accessibility demonstrated a stronger association with residents’ mental health [111]. A number of studies have also shown that accessibility, openness, and safety of green space amenities are associated with reduced stress, increased social interaction, and lower prevalence of mental illness [112,113,114]. This shift may be contextualized within the profound transformation in social lifestyles during the COVID-19 pandemic. Lockdown measures, restrictions on indoor socialization, transitions to remote work, and heightened mental health stress collectively reshaped daily life during the pandemic. These changes contributed to a growing need and increased opportunities for green space utilization. For example, a previous study suggests that working from home during the pandemic enhanced individuals’ access to nearby green spaces and elevated public appreciation for their value [115,116]. This transformation has not only altered the thematic focus of research but has also indirectly reinforced the dominance of perception and self-report methods in evidence production.

Urban residents’ perceptions of green space functions transitioned from a focus on “accessibility” to “perceived experience”, emphasizing subjective feelings of comfort, safety, and aesthetic quality. These perceptual factors were increasingly identified as key correlates of both usage behavior and psychological outcomes. This shift aligns with the theory of Situated Green Value [117], which posits that the value of green space is no longer rooted solely in its physical attributes, but rather in a subjective construct shaped by emotional states, lived experiences, and social contexts.

Additionally, the prominence of the “Social and Recreational Usability” dimension can be interpreted as a response to the “weak-tie restoration mechanism.” Even under conditions of social distancing, individuals continued to derive social validation and emotional resonance through the dynamics of “being seen” and “seeing others” in public green spaces [118]. This phenomenon is reflected in a study on shifting urban housing preferences [119], showing that individuals increasingly favor residences near green spaces or with private gardens for psychological security and social interaction.

Therefore, it can be argued that the COVID-19 pandemic not only reignited public awareness of the mental health value of the natural environment but also prompted a paradigm shift in academic research—from traditional, objective measures of green space, such as cumulative area, NDVI, and canopy cover, toward a more subjective focus on perceptual experiences and social functionality. This evolving research trajectory suggests that future urban green space planning might benefit from incorporating user-centered perspectives, emphasizing individuals’ subjective experiences, emotional needs, lifestyles, and latent demands. Such an approach may be valuable for enhancing the psychological resilience and equity of green space utilization. Consequently, this pandemic has spurred both a surge in research output and the convergence of research methodologies grounded in subjective perception. This pattern reflects how situational pressures may influence cognitive preferences within scientific inquiry.

### 5.2. From Pathology to Positive Mental Health: Evolution of Theoretical Orientation

The results indicate that research on “Emotional Disorders” and “Stress and Trauma” remains substantial. However, in recent years, “Well-Being and Resilience” has emerged as the most prominent theme in urban green space and mental health research, accounting for nearly 80% of all studies and reaching a historical peak in 2024. This trend reflects a paradigmatic shift in focus, from the traditional pathology-centered approach to an emphasis on the maintenance and promotion of positive psychological states. This shift also marks a profound transformation in research thinking, moving from diagnosing psychological issues to promoting psychological resources. Ultimately, this may reshape how mental health is defined and measured.

This shift is consistent with several recent studies. For instance, it has been emphasized that the psychological value of green spaces extends beyond alleviating symptoms such as anxiety and depression, encompassing the enhancement of individuals’ capacity to adapt to environmental challenges, specifically, the development of psychosocial resilience [120]. Similarly, it has been argued that in the post-pandemic era, urban planning could prioritize the systemic role of green spaces in fostering everyday well-being, rather than treating them merely as supplementary interventions during health crises [121].

However, a comparison of the number of studies does not exhibit a zero-sum relationship of pathological and positive research directions. This indicates that the academic community is increasingly adopting a more holistic mental health framework, one that conceptualizes green spaces as cross-cutting resources for supporting multidimensional mental well-being, rather than as singular therapeutic interventions. This integrative trend also aligns with the “dual-track strategy for green health”: on one track, green spaces are employed to mitigate and treat emotional disorders; on the other, they are embedded into everyday environments to cultivate a prevention-oriented, positive psychological ecology [122,123].

Therefore, we suggest that future research and policy advance the “dual-track strategy”. Efforts could continue to deepen our understanding of the therapeutic mechanisms of green spaces in addressing pathological psychological conditions (e.g., depression, anxiety, trauma). In addition, there is a potential to rigorously explore how green spaces can foster positive psychological outcomes by enhancing resilience, a sense of belonging, and life satisfaction. This approach will facilitate a transition from the paradigm of “green spaces for psychological disorders” to one of “urban ecosystems for positive mental health.” However, this evolution of conceptual frameworks would benefit from corresponding methodological innovations to avoid reinforcing the self-reported circular reasoning between theoretical and measurement preferences.

### 5.3. Methodological Concentration and Measurement Bias

Based on quantitative research findings, this study identifies a methodological bias toward self-report measures over objective measurement tools. This study reveals that current research on urban green spaces and mental health remains heavily reliant on subjective self-report scales, particularly within “Well-Being and Resilience”, indicating a pronounced methodological imbalance. While subjective scales offer advantages in terms of ease of administration and the ability to capture individuals’ experiential perceptions and psychological responses, they also have notable limitations. In particular, establishing causality, identifying underlying mechanisms, and enabling cross-cultural comparisons may compromise the external validity and policy applicability of the findings.

Although a limited number of studies have begun to incorporate objective indicators from physiological or behavioral domains, their overall representation remains minimal and is primarily concentrated within “Well-Being and Resilience” and “Emotional Disorder”. For instance, one study employed objective measures such as blood pressure, heart rate, and heart rate variability (HRV) to compare the stress-relieving effects of walking in urban green spaces versus non-green environments [124]. Another study used HRV to assess the physiological and psychological relaxation effects of viewing small-scale urban green spaces (biotopes) among depressed outpatients [125]. In addition, a further study applied HRV in combination with salivary cortisol biomarkers to examine the stress-relieving benefits of walking in green space environments [126].

However, such multi-source integration methods remain in the early stages of exploration and have yet to be widely implemented across diverse population samples. The limitations in behavioral data collection are particularly pronounced. For example, within studies addressing “Sleep and Behavioral Functioning”, results using objective recordings appear lower than what would be theoretically anticipated. This shortfall is likely associated with the limited standardization and adoption of behavioral tracking technologies (e.g., smart bracelets, gait recognition systems) within current urban health research practices.

The integration of objective measures, such as physiological recordings and behavioral tracking, presents several limitations related to ethical, financial, and technical factors. First, the collection of physiological and behavioral data is often subject to strict privacy considerations and ethical approval processes [127] creating substantial barriers for large-scale or multi-city studies. Moreover, the high costs associated with equipment procurement and maintenance, the requirement for specialized technical training, and the sensitivity of physiological data, which necessitates complex data management protocols, further contribute to increased financial and logistical burdens [128,129]. Additionally, the variable or delayed physiological responses to green space exposure, such as changes in cortisol levels or heart rate variability (HRV), which may require hours to manifest a stable effect following contact with natural environments [130,131], may have led many researchers to favor the use of subjective self-report tools that offer immediate feedback (e.g., PANAS, WHO-5).

Furthermore, the present study suggests that the adoption of objective measurement techniques imposes greater demands on interdisciplinary collaboration, while most existing research remains rooted primarily in psychology or urban planning. Such research often lacks the capacity to systematically integrate physiological, behavioral, and environmental datasets in cooperation with disciplines such as medicine and biomedical science. This path dependency in methodology appears to be associated with feasibility-driven study designs and disciplinary conventions, rather than being driven exclusively by considerations of validity and representativeness.

On the other hand, the enduring reliance on traditional psychological assessment methods, such as self-report questionnaires, in the fields of environmental psychology and public health continues to offer notable advantages. Their ease of administration, high degree of standardization, and suitability for large sample sizes have contributed to their widespread adoption. Additional benefits include their replicability, simplicity in implementation, and the facilitation of cross-study and cross-sample comparisons through the use of standardized instruments.

Overall, the economic, technological, and ethical barriers associated with objective measurement instruments, combined with the entrenched reliance on self-assessment questionnaires, constitute a significant source of structural bias in measurement approaches within green space and psychological research. This methodological bias may compromise the ecological validity of study findings. Self-reported data are particularly vulnerable to recall bias, social desirability effects, and cultural framing, especially in cross-national or longitudinal studies [132]. Due to the lack of validation through objective indicators such as heart rate variability and sleep patterns, the causal mechanisms between green space exposure and mental health remain less frequently examined with reliable and specific evidence [133]. This limitation also constrains the applicability of research findings to green space policy making and design practices.

Furthermore, this path dependency in measurement practices has exacerbated knowledge redundancy and methodological conservatism in academic research. It also impedes cross-modal data integration and innovation in mechanism discovery [96,122]. This misalignment risks a potential disconnect between the evolving knowledge base and socially prioritized public health concerns. Instead, these patterns indicate that current research evidence is shaped more by feasibility and accessibility than by representativeness and clinical relevance. The next section will broaden the scope from measurement-level asymmetry to sampling-level inequality, revealing how epistemological biases manifest in population coverage.

### 5.4. Population-Level Bias and Sampling Inequity

Beyond methodological imbalances, the findings also reveal structural inequalities in population representation, indicating that potential gaps in the evidence base are also evident through the selection of research subjects. The findings of this study indicate a significant imbalance in sample structures within current research on urban green spaces and mental health, with the majority of studies concentrating on healthy adults, university students, and general urban populations. In contrast, children, clinical patients, older adults, and specific occupational groups are comparatively underrepresented.

In light of this population coverage bias, we argue that the widespread reliance on large-sample questionnaires, such as those targeting healthy adults or general urban populations, is shaped by multiple factors in urban green space and mental health research. These include the convenience of data collection as well as the emphasis on statistical significance and the generalizability of findings in public health contexts. Policymakers have increasingly emphasized the demand for “evidence of broad applicability” [134]. Similarly, it has been highlighted that sample size is a critical variable in public health research, particularly within heterogeneous urban populations, as it helps mitigate bias, enhance reliability, and strengthen causal inference between variables [135]. However, to overcome the resulting geographic and resource-related disparities, future strategies could move beyond convenience-based sampling. This entails allocating targeted funding for recruitment in under-resourced regions and incentivizing multi-site collaborations to ensure the inclusion of geographically dispersed and vulnerable populations.

However, we suggest that this strong emphasis on sample size may inadvertently favor highly accessible groups (e.g., college students, healthy adults), thereby further exacerbating sample structure imbalances. For instance, some studies observe that researchers often make strategic trade-offs between sample acquisition and resource allocation, and that such population selection based on implementation convenience may enhance operational efficiency [136,137]. This approach, however, has the potential to undermine the interpretability and fairness of psychological insights related to marginalized groups.

Moreover, experimental studies, another frequently employed study design, often rely on college students as convenience samples due to the need for sample control, leading to a tendency toward “campus centrism.” In contrast, special populations such as children and clinical patients are more frequently studied through qualitative methods, owing to challenges such as limited accessibility, complex ethical approval processes, and restricted communicative capacity. While this methodological “strategic choice” may be pragmatically justified, it nevertheless entails compromises regarding sample characteristics, ultimately reducing the comparability and interpretive breadth of the research findings.

This structural bias may significantly constrain our understanding of the mental health effects of urban green spaces across diverse social contexts, particularly in evaluating their role in addressing mental health disparities. Recent studies have also highlighted the impact of this issue on the explanatory power of empirical evidence. It has been observed that marginalized populations exhibit distinct patterns in their use of urban green spaces and in their psychological responses [138]. Neglecting this heterogeneity leads to a systematic underestimation of the restorative potential of urban environments. Similarly, a study in an urban community in Beijing found that perceptions of barriers and experiences of social isolation in green space use were significantly higher among chronically ill individuals compared to healthy residents. Moreover, the psychological benefits among these groups were more contingent upon accessible design and inclusive spatial configurations [139].

To advance equity in this field, future research would benefit from prioritizing inclusive study designs that adequately represent diverse populations, including children, older adults, patients, and low-income groups. This could be achieved by improving sample accessibility through multicenter surveys and collaborations with healthcare systems. This can also be achieved by developing mixed-method approaches tailored to vulnerable populations, such as assisted interviews, behavioral observation, and wearable monitoring technologies, to enhance data quality and interpretive depth. Addressing these parochial sample structures may help render urban green space policies more inclusive and conducive to health equity.

Mitigating epistemic inequity requires not only a diversity of methodologies but also an inclusive population structure, especially for the elderly, children, and people with physical or mental disabilities, to ensure that future research evidence better reflects the diversity of urban mental health outcomes.

### 5.5. Limitations

This systematic review has several limitations that should be acknowledged. First, the literature search was restricted to the Web of Science database, and included only peer-reviewed articles published in English between 2004 and 2024. This single-database strategy excluded other major repositories, such as Scopus and PubMed, which index a significant volume of relevant literature in public health, psychology, and environmental research. Consequently, relevant studies published in other languages, indexed in these databases, or available as grey literature (e.g., technical reports, dissertations) may have been missed, introducing potential language, indexing, and publication bias. From an epistemic equity perspective, these restrictions may further privilege Anglophone and high-income country scholarship and underrepresent local, non-English, and regionally indexed evidence; therefore, the patterns identified in this review should be interpreted as characterizing the currently dominant English-language evidence base. These restrictions likely contributed to the geographic imbalances reported in Supplementary Table S1. For instance, the substantial underrepresentation of studies from South America and Africa could be partly due to the exclusion of non-English local journals or regional databases (e.g., SciELO, LILACS), rather than reflecting an actual dearth of research activity in these regions.

Second, the review applied stringent exclusion criteria, omitting review articles, conference proceedings, and non-peer-reviewed publications. While this enhances methodological rigor, it may be associated with the exclusion of valuable insights and comprehensive syntheses. Third, despite implementing a dual-reviewer process for screening and data extraction, a degree of subjectivity remains inevitable, particularly in the evaluation of studies with incomplete or ambiguous reporting. Although discrepancies were resolved through consensus, this may still influence the consistency and reproducibility of the findings. Fourth, substantial heterogeneity among the included studies in terms of design, outcome measures, and reporting standards precluded a formal risk of bias assessment or a quantitative meta-analysis. Although this limits our ability to evaluate the internal validity of individual studies or aggregate statistical power, this descriptive approach was necessary to accommodate the broad scope of the review and to focus on identifying structural methodological biases and gaps in population representation (epistemic equity) across the 235 studies.

Furthermore, incomplete reporting in some primary studies, such as missing data on sample characteristics, effect sizes, or measurement instruments, may have constrained the comprehensiveness of the synthesis and the depth of interpretation. These limitations reduce the generalizability and applicability of the findings across diverse contexts.

Finally, this review did not conduct a formal assessment of reporting bias, as no quantitative synthesis or meta-analysis was performed. However, potential reporting biases may exist due to publication bias (e.g., studies with null results being underreported) and language restrictions (English-only publications). No formal GRADE-based assessment of evidence certainty was conducted, as this review synthesized heterogeneous and non-comparable studies using a descriptive analytical approach. Nevertheless, the overall confidence in the findings can be regarded as moderate, given the consistent patterns observed across multiple independent studies.

## 6. Conclusions

This systematic review synthesizes the international literature on the psychological impacts of urban public green spaces, highlighting the paradigm shifts and methodological tendencies that have emerged over the past two decades, particularly after the COVID-19 pandemic. Our analysis identifies a clear transition in research emphasis from the use of objective indicators and spatial metrics of green space to the exploration of individual subjective experiences. In parallel, the field has moved from a pathology-centered paradigm toward a dual-track approach that integrates both therapeutic and preventive dimensions of mental health.

From a methodological perspective, the current body of evidence shows a consistent pattern of epistemic bias. Research feasibility and data accessibility have strongly influenced methodological choices. They shape not only how green spaces and mental health are studied, but also whose experiences are represented. Most studies rely on cross-sectional, self-reported data collected from healthy adults and university students, while experimental, longitudinal, or physiologically validated approaches remain limited. These structural imbalances weaken the generalizability, inclusiveness, and practical relevance of existing findings, limiting their contribution to equitable public-health interventions.

Achieving epistemic equity requires methodological pluralism that combines experimental, physiological, and participatory approaches with inclusive sampling strategies extending beyond healthy adult and convenience groups. To proceed in this direction, future research should prioritize longitudinal and experimental designs, multimodal data collection, and close interdisciplinary collaboration among urban planning, public health, and medical fields. Such diversification is essential for producing robust and actionable evidence that can inform inclusive urban planning and promote mental-health equity in an increasingly urbanized world.

## Figures and Tables

**Table 1:** Mapping between study design, population sampling, and psychometric measures in urban green space and mental health research (356 combinations from 235 studies).

Study Design	Self-Report Measures				Clinician-Rated Psychometric Measures				Physiological and Biochemical Indicators				Behavioral and Observational Measures			
	CA	AV	CH	IC	CA	AV	CH	IC	CA	AV	CH	IC	CA	AV	CH	IC
Cross-sectional study	99	22	3	9	34	12	0	3	1	1	0	2	11	1	0	0
Longitudinal study	8	2	0	1	5	2	0	0	2	0	0	0	2	0	0	0
Experimental research	27	1	1	14	16	0	0	11	18	1	1	10	1	0	0	0
Quasi-experimental study	4	0	2	0	2	0	0	0	3	0	0	0	1	0	0	0
Case Study	5	1	0	1	1	0	0	0	1	0	0	0	0	0	0	0
Qualitative descriptive design	7	1	1	1	1	0	0	1	0	0	0	1	0	0	1	0
Frequency range																
1–5																
6–10																
11–15																
16–20																
21–30																
31–40																
41~																

Note: The table presents all unique combinations of study design, psychometric measures, and population sampling categories. If a single study included multiple participant groups or psychometric measures, each relevant combination was counted in all corresponding cells. Abbreviations: CA, Community-dwelling adult populations; AV, Age-specific vulnerable populations; CH, Clinical or high-risk populations; IC, Institutional or convenience populations. Color intensity indicates the frequency of observed combinations, with darker blue representing higher frequencies.

## Data Availability

All data analyzed in this review were derived from previously published articles. No new datasets or analytical code were generated. The Supplementary Materials include the PRISMA flow diagram, data extraction tables, and the completed PRISMA 2020 Checklist. These materials are available from the corresponding author upon reasonable request (r.shimoda@chiba-u.jp).

## Supplementary Materials

The following supporting information can be downloaded at: https://www.mdpi.com/article/10.3390/ijerph23020218/s1, Figure S1: Number of publications per year; Figure S2: Annual number of studies on mental health dimensions in urban green space and mental health research (2004–2024); File S1: PRISMA 2020 checklist (Reference [34] is cited in the supplementary File S1); Table S1: Web of Science topic filters excluded at the screening stage and number of records removed in each category; Table S2: Extracted dataset of the 235 included studies (References [13,14,35,46,49,54,57,58,61,62,64,65,66,68,69,70,71,72,73,74,75,78,83,89,90,91,107,110,129,140,141,142,143,144,145,146,147,148,149,150,151,152,153,154,155,156,157,158,159,160,161,162,163,164,165,166,167,168,169,170,171,172,173,174,175,176,177,178,179,180,181,182,183,184,185,186,187,188,189,190,191,192,193,194,195,196,197,198,199,200,201,202,203,204,205,206,207,208,209,210,211,212,213,214,215,216,217,218,219,220,221,222,223,224,225,226,227,228,229,230,231,232,233,234,235,236,237,238,239,240,241,242,243,244,245,246,247,248,249,250,251,252,253,254,255,256,257,258,259,260,261,262,263,264,265,266,267,268,269,270,271,272,273,274,275,276,277,278,279,280,281,282,283,284,285,286,287,288,289,290,291,292,293,294,295,296,297,298,299,300,301,302,303,304,305,306,307,308,309,310,311,312,313,314,315,316,317,318,319,320,321,322,323,324,325,326,327,328,329,330,331,332,333,334,335,336,337,338,339,340,341,342,343,344,345] are cited in the supplementary Table S2); Table S3: Continents and countries where studies have been conducted on the mental health role of urban public green spaces; Text S1: Retrospective review protocol; Text S2: Codebook.

## References

[1] Peen J., Schoevers R.A., Beekman A.T., Dekker J. (2010). The current status of urban-rural differences in psychiatric disorders. Acta Psychiatr Scand.

[2] Vassos E., Pedersen C.B., Murray R.M., Collier D.A., Lewis C.M. (2012). Meta-analysis of the association of urbanicity with schizophrenia. Schizophr Bull.

[3] van den Bosch M., Sang Å. (2016). Urban Green Spaces and Health—A Review of Evidence.

[4] Liu Z., Chen X., Cui H., Ma Y., Gao N., Li X., Meng X., Lin H., Abudou H., Guo L. (2023). Green space exposure on depression and anxiety outcomes: A meta-analysis. Environ. Res..

[5] Twohig-Bennett C., Jones A. (2018). The health benefits of the great outdoors: A systematic review and meta-analysis of greenspace exposure and health outcomes. Environ. Res..

[6] White M.P., Alcock I., Grellier J., Wheeler B.W., Hartig T., Warber S.L., Bone A., Depledge M.H., Fleming L.E. (2019). Spending at least 120 minutes a week in nature is associated with good health and wellbeing. Sci. Rep..

[7] Lopez-Haro J., Gómez-Chávez L.F.J., Pelayo-Zavalza A.R., Gómez-Varela J.F. (2024). Association between Active Use of Urban Green Spaces and Well-Being in Adults Aged 18-65 Years: A Systematic Review. J. Health Pollut..

[8] Huang W., Lin G. (2023). The relationship between urban green space and social health of individuals: A scoping review. Urban For. Urban Green..

[9] Li H., Browning M.H.E.M., Dzhambov A.M., Zhang G., Cao Y. (2022). Green Space for Mental Health in the COVID-19 Era: A Pathway Analysis in Residential Green Space Users. Land.

[10] Xie J., Luo S., Furuya K., Sun D. (2020). Urban Parks as Green Buffers During the COVID-19 Pandemic. Sustainability.

[11] Lachowycz K., Jones A.P. (2011). Greenspace and obesity: A systematic review of the evidence. Obes. Rev..

[12] Markevych I., Schoierer J., Hartig T., Chudnovsky A., Hystad P., Dzhambov A.M., de Vries S., Triguero-Mas M., Brauer M., Nieuwenhuijsen M.J. (2017). Exploring pathways linking greenspace to health: Theoretical and methodological guidance. Environ. Res..

[13] Shanahan D.F., Bush R., Gaston K.J., Lin B.B., Dean J., Barber E., Fuller R.A. (2016). Health Benefits from Nature Experiences Depend on Dose. Sci. Rep..

[14] Wolch J.R., Byrne J., Newell J.P. (2014). Urban green space, public health, and environmental justice: The challenge of making cities ‘just green enough’. Landsc. Urban Plan..

[15] Rigolon A. (2016). A complex landscape of inequity in access to urban parks: A literature review. Landsc. Urban Plan..

[16] Zhao Y., Gong P. (2024). Optimal site selection strategies for urban parks green spaces under the joint perspective of spatial equity and social equity. Front. Public Health.

[17] Jennings V., Rigolon A., Thompson J., Murray A., Henderson A., Gragg R.S. (2024). The Dynamic Relationship between Social Cohesion and Urban Green Space in Diverse Communities: Opportunities and Challenges to Public Health. Int. J. Environ. Res. Public Health.

[18] Das D.K. (2025). Exploring the Intersection of Environmental Justice and Urban Green Space Planning: A Systematic Review. Urban Sci..

[19] Dell’Ovo M., Datola G., Maiullari D., Oppio A., Schretzenmayr M. (2026). Green Gentrification: A Literature Review of Trends, Challenges, and Research Opportunities. Computational Science and Its Applications–ICCSA 2025 Workshops.

[20] Borderon M., Best K.B., Bailey K., Hopping D.L., Dove M., Cervantes de Blois C.L. (2021). The risks of invisibilization of populations and places in environment-migration research. Humanit. Soc. Sci. Commun..

[21] Freymueller J., Schmid H.-L., Senkler B., Lopez Lumbi S., Zerbe S., Hornberg C., McCall T. (2024). Current methodologies of greenspace exposure and mental health research—A scoping review. Front. Public Health.

[22] Reyes-Riveros R., Altamirano A., De La Barrera F., Rozas-Vásquez D., Vieli L., Meli P. (2021). Linking public urban green spaces and human well-being: A systematic review. Urban For. Urban Green..

[23] Fricker M. (2007). Epistemic Injustice: Power and the Ethics of Knowing.

[24] Dotson K. (2014). Conceptualizing Epistemic Oppression. Soc. Epistemol..

[25] Braveman P., Gruskin S. (2003). Defining equity in health. J. Epidemiol. Community Health.

[26] Schlosberg D. (2007). Defining Environmental Justice: Theories, Movements, and Nature.

[27] Alvesson M., Sandberg J. (2011). Generating Research Questions Through Problematization. Acad. Manag. Rev..

[28] Beute F., Marselle M.R., Olszewska-Guizzo A., Andreucci M.B., Lammel A., Davies Z.G., Glanville J., Keune H., O’Brien L., Remmen R. (2023). How do different types and characteristics of green space impact mental health? A scoping review. People Nat..

[29] Chen K., Zhang T., Liu F., Zhang Y., Song Y. (2021). How Does Urban Green Space Impact Residents’ Mental Health: A Literature Review of Mediators. Int. J. Environ. Res. Public Health.

[30] Legg R., Kabisch N. (2024). The effects of allergenic pollen in green space on mental health, behaviour and perceptions: A systematic review. Urban For. Urban Green..

[31] Gianfredi V., Buffoli M., Rebecchi A., Croci R., Oradini-Alacreu A., Stirparo G., Marino A., Odone A., Capolongo S., Signorelli C. (2021). Association between Urban Greenspace and Health: A Systematic Review of Literature. Int. J. Environ. Res. Public Health.

[32] Wu X., Shen Y.S., Cui S. (2023). Global Trends in Green Space and Senior Mental Health Studies: Bibliometric Review. Int. J. Environ. Res. Public Health.

[33] Aghabozorgi K., van der Jagt A., Bell S., Smith H. (2024). How university blue and green space affect students’ mental health: A scoping review. Urban For. Urban Green..

[34] Page M.J., McKenzie J.E., Bossuyt P.M., Boutron I., Hoffmann T.C., Mulrow C.D., Shamseer L., Tetzlaff J.M., Akl E.A., Brennan S.E. (2021). The PRISMA 2020 statement: An updated guideline for reporting systematic reviews. BMJ.

[35] Nieuwenhuijsen M.J., Dadvand P., Márquez S., Bartoll X., Barboza E.P., Cirach M., Borrell C., Zijlema W.L. (2022). The evaluation of the 3-30-300 green space rule and mental health. Environ. Res..

[36] Green Social Prescribing Improves Your Mental Health. https://socialprescribingacademy.org.uk/resources/green-social-prescribing-improves-your-mental-health/?utm_source=chatgpt.com.

[37] Darcy P.M., Armitt H., Hurd A., Paton L.W., White P.C.L., Coventry P.A. (2025). Green Social Prescribing: A Before and After Evaluation of a Novel Community-Based Intervention for Adults Experiencing Mental Health Problems. Health Soc. Care Community.

[38] Cooper M., Avery L., Scott J., Ashley K., Jordan C., Errington L., Flynn D. (2022). Effectiveness and active ingredients of social prescribing interventions targeting mental health: A systematic review. BMJ Open.

[39] Social Benefits of Green Infrastructure. https://www.epa.gov/green-infrastructure/social-benefits-green-infrastructure.

[40] 10-Minute Walk. https://10minutewalk.org/.

[41] Governing Urban Parks and the 10-Minute Challenge. Abbreviation 2017. https://www.governing.com/gov-institute/voices/col-urban-parks-10-minute-challenge.html.

[42] NParks Introduces New Parks for Health Framework Aimed at Leveraging Existing Parks and Therapeutic Landscapes to Boost Community Well-Being. https://www.nparks.gov.sg/news/news-detail/nparks-introduces-new-parks-for-health-framework-aimed-at-leveraging-existing-parks-and-therapeutic-landscapes-to-boost-community-well-being.

[43] Planning a City for Health and Well-Being. https://isomer-user-content.by.gov.sg/50/102dfebc-79f1-4800-8dfa-50e246796683/uss-planning-a-city-for-health-and-well-being.pdf.

[44] Healthy China Action-Healthy Environment Promotion Action Implementation Program (2025–2030). https://www.gov.cn/zhengce/zhengceku/202508/content_7035320.htm.

[45] African Union Green Recovery Action Plan. https://faolex.fao.org/docs/pdf/au225399.pdf.

[46] Nutsford D., Pearson A.L., Kingham S. (2013). An ecological study investigating the association between access to urban green space and mental health. Public Health.

[47] Oh R.R.Y., Fielding K.S., Chang C.C., Nghiem L.T.P., Tan C.L.Y., Quazi S.A., Shanahan D.F., Gaston K.J., Carrasco R.L., Fuller R.A. (2021). Health and Wellbeing Benefits from Nature Experiences in Tropical Settings Depend on Strength of Connection to Nature. Int. J. Environ. Res. Public Health.

[48] Bergefurt L., Kemperman A., van den Berg P., Borgers A., van der Waerden P., Oosterhuis G., Hommel M. (2019). Loneliness and Life Satisfaction Explained by Public-Space Use and Mobility Patterns. Int. J. Environ. Res. Public Health.

[49] Roe J.J., Thompson C.W., Aspinall P.A., Brewer M.J., Duff E.I., Miller D., Mitchell R., Clow A. (2013). Green Space and Stress: Evidence from Cortisol Measures in Deprived Urban Communities. Int. J. Environ. Res. Public Health.

[50] Jiang B., Chang C.-Y., Sullivan W.C. (2014). A dose of nature: Tree cover, stress reduction, and gender differences. Landsc. Urban Plan..

[51] Jennings V., Bamkole O. (2019). The Relationship between Social Cohesion and Urban Green Space: An Avenue for Health Promotion. Int. J. Environ. Res. Public Health.

[52] Wood L., Hooper P., Foster S., Bull F. (2017). Public green spaces and positive mental health—Investigating the relationship between access, quantity and types of parks and mental wellbeing. Health Place.

[53] Ojala A., Korpela K., Tyrväinen L., Tiittanen P., Lanki T. (2019). Restorative effects of urban green environments and the role of urban-nature orientedness and noise sensitivity: A field experiment. Health Place.

[54] Wang X., Rodiek S., Chengzhao W., Chen Y., Li Y. (2016). Stress Recovery and Restorative Effects of Viewing Different Urban park Scenes in Shanghai, China. Urban For. Urban Green..

[55] Olszewska-Guizzo A., Sia A., Fogel A., Ho R. (2022). Features of urban green spaces associated with positive emotions, mindfulness and relaxation. Sci. Rep..

[56] Orstad S.L., Szuhany K., Tamura K., Thorpe L.E., Jay M. (2020). Park Proximity and Use for Physical Activity among Urban Residents: Associations with Mental Health. Int. J. Environ. Res. Public Health.

[57] Park K.H. (2022). Analysis of Urban Forest Healing Program Expected Values, Needs, and Preferred Components in Urban Forest Visitors with Diseases: A Pilot Survey. Int. J. Environ. Res. Public Health.

[58] Pope D., Tisdall R., Middleton J., Verma A., van Ameijden E., Birt C., Macherianakis A., Bruce N.G. (2018). Quality of and access to green space in relation to psychological distress: Results from a population-based cross-sectional study as part of the EURO-URHIS 2 project. Eur. J. Public Health.

[59] Pasanen T.P., Tyrväinen L., Korpela K.M. (2014). The relationship between perceived health and physical activity indoors, outdoors in built environments, and outdoors in nature. Appl. Psychol. Health Well Being.

[60] Grigsby-Toussaint D.S., Turi K.N., Krupa M., Williams N.J., Pandi-Perumal S.R., Jean-Louis G. (2015). Sleep insufficiency and the natural environment: Results from the US Behavioral Risk Factor Surveillance System survey. Prev. Med..

[61] Mokhtar D., Abdul Aziz N.A., Mariapan M. (2018). Physiological and Psychological Health Benefits of Urban Green Space in Kuala Lumpur: A comparison between Taman Botani Perdana and Jalan Bukit Bintang. Pertanika J. Soc. Sci. Humanit..

[62] Tomasso L.P., Spengler J.D., Catalano P.J., Chen J.T., Laurent J.G.C. (2023). In situ psycho-cognitive assessments support self-determined urban green exercise time. Urban For. Urban Green..

[63] Wolf K.L., Senturia K., Simmons C., Hafferty K., Faino A.V., Garrett K.A., Tandon P.S. (2024). Participatory Design for Community Schoolyards: Mixed Methods Reveal Positive Engagement Despite Measures Limitations. Ecopsychology.

[64] Mayen Huerta C., Utomo A. (2021). Evaluating the association between urban green spaces and subjective well-being in Mexico city during the COVID-19 pandemic. Health Place.

[65] Lau K.K.-L., Yung C.C.-Y., Tan Z. (2021). Usage and perception of urban green space of older adults in the high-density city of Hong Kong. Urban For. Urban Green..

[66] Honold J., Beyer R., Lakes T., van der Meer E. (2012). Multiple environmental burdens and neighborhood-related health of city residents. J. Environ. Psychol..

[67] Turunen A.W., Halonen J., Korpela K., Ojala A., Pasanen T., Siponen T., Tiittanen P., Tyrväinen L., Yli-Tuomi T., Lanki T. (2023). Cross-sectional associations of different types of nature exposure with psychotropic, antihypertensive and asthma medication. Occup. Environ. Med..

[68] Zhang Y., Van den Berg A.E., Van Dijk T., Weitkamp G. (2017). Quality over Quantity: Contribution of Urban Green Space to Neighborhood Satisfaction. Int. J. Environ. Res. Public Health.

[69] Hijazi B., Tirosh E., Chudnovsky A., Saadi D., Schnell I. (2023). The short term adaptation of the autonomic nervous systems (ANS) by type of urban environment and ethnicity. Environ. Res..

[70] Huang C., Wei F., Qiu S., Cao X., Chen L., Xu J., Gao J., Lin Q. (2023). Interpreting regenerated post-industrial lands as green spaces: Comparing public perceptions of post-industrial landscapes using human factor design framework. Ecol. Indic..

[71] Lu X., Peng Y., Song S., Wang H., Yin Y., Wang J.-j. (2024). The nose cooperates with the eyes: The independent and interactive effects of vision and olfaction on the perceived restorativeness of a Metasequoia walkway. Urban For. Urban Green..

[72] Tao T., Shi Y., Yang Q., Li S., Guo X., Pei X. (2023). Characteristics of hospitalized patients with depression and their relationship with the surrounding environment: A study in a class 3A hospital in Shanghai. Curr. Psychol..

[73] Wang Z., Fu H., Jian Y., Qureshi S., Jie H., Wang L. (2022). On the comparative use of social media data and survey data in prioritizing ecosystem services for cost-effective governance. Ecosyst. Serv..

[74] Huang S., Zhu J., Zhai K., Wang Y., Wei H., Xu Z., Gu X. (2022). Do Emotional Perceptions of Visible Greeneries Rely on the Largeness of Green Space? A Verification in Nanchang, China. Forests.

[75] Krenichyn K. (2006). ‘The only place to go and be in the city’: Women talk about exercise, being outdoors, and the meanings of a large urban park. Health Place.

[76] Ruijsbroek A., Droomers M., Kruize H., Van Kempen E., Gidlow C.J., Hurst G., Andrusaityte S., Nieuwenhuijsen M.J., Maas J., Hardyns W. (2017). Does the Health Impact of Exposure to Neighbourhood Green Space Differ between Population Groups? An Explorative Study in Four European Cities. Int. J. Environ. Res. Public Health.

[77] Patwary M.M., Bardhan M., InAn H.E., Browning M.H.E.M., Disha A.S., Haque Z., Helmy M., Ashraf S., Dzhambov A.M., Shuvo F.K. (2024). Exposure to urban green spaces and mental health during the COVID-19 pandemic: Evidence from two low and lower-middle-income countries. Front. Public Health.

[78] Spano G., Nobile F., Giannico V., Elia M., Michelozzi P., Bosco A., Dadvand P., Sanesi G., Stafoggia M. (2023). Two- and three-dimensional indicators of green and grey space exposure and psychiatric conditions and medicine use: A longitudinal study in a large population-based Italian cohort. Environ. Int..

[79] Xie B., Lu Y., Zheng Y. (2022). Casual evaluation of the effects of a large-scale greenway intervention on physical and mental health: A natural experimental study in China. Urban For. Urban Green..

[80] Zhang X., Qi J., Lin E.S., Tan P.Y., Ho R., Sia A., Song X.P., Waykool R., Olszewska-Guizzo A. (2024). Towards healthy cities: Modeling restorative potential of urban environments by coupling LiDAR-derived 3D metrics with panorama-based online survey. Environ. Impact Assess. Rev..

[81] Wei H., Zhang J., Xu Z., Hui T., Guo P., Sun Y. (2022). The association between plant diversity and perceived emotions for visitors in urban forests: A pilot study across 49 parks in China. Urban For. Urban Green..

[82] Bressane A., Silva M.B., Goulart A.P., Medeiros L.C. (2024). Understanding How Green Space Naturalness Impacts Public Well-Being: Prospects for Designing Healthier Cities. Int. J. Environ. Res. Public Health.

[83] Schertz K.E., Sachdeva S., Kardan O., Kotabe H.P., Wolf K.L., Berman M.G. (2018). A thought in the park: The influence of naturalness and low-level visual features on expressed thoughts. Cognition.

[84] Weimann H., Björk J., Håkansson C. (2019). Experiences of the Urban Green Local Environment as a Factor for Well-Being among Adults: An Exploratory Qualitative Study in Southern Sweden. Int. J. Environ. Res. Public Health.

[85] van den Bosch M.A., Östergren P.O., Grahn P., Skärbäck E., Währborg P. (2015). Moving to Serene Nature May Prevent Poor Mental Health--Results from a Swedish Longitudinal Cohort Study. Int. J. Environ. Res. Public Health.

[86] Nordbø E.C.A., Raanaas R.K., Nordh H., Aamodt G. (2020). Disentangling how the built environment relates to children’s well-being: Participation in leisure activities as a mediating pathway among 8-year-olds based on the Norwegian Mother and Child Cohort Study. Health Place.

[87] Pasanen T., Johnson K., Lee K., Korpela K. (2018). Can Nature Walks With Psychological Tasks Improve Mood, Self-Reported Restoration, and Sustained Attention? Results From Two Experimental Field Studies. Front. Psychol..

[88] Derose K., Wallace D., Han B., Cohen D. (2021). Effects of park-based interventions on health-related outcomes: A systematic review. Prev. Med..

[89] Qi Y., Chen Q., Lin F., Liu Q., Zhang X., Guo J., Qiu L., Gao T. (2022). Comparative study on birdsong and its multi-sensory combinational effects on physio-psychological restoration. J. Environ. Psychol..

[90] Zheng Y., Lin T., Hamm N.A., Liu J., Zhou T., Geng H., Zhang J., Ye H., Zhang G., Wang X. (2024). Quantitative evaluation of urban green exposure and its impact on human health: A case study on the 3–30-300 green space rule. Sci. Total Environ..

[91] Jiang X., Hu Y., Larsen L., Chang C.-Y., Sullivan W.C. (2023). Impacts of urban green infrastructure on attentional functioning: Insights from an fMRI study. Front. Psychol..

[92] Henrich J., Heine S.J., Norenzayan A. (2010). The weirdest people in the world?. Behav. Brain Sci..

[93] Koutsos T.M., Menexes G.C., Dordas C.A. (2019). An efficient framework for conducting systematic literature reviews in agricultural sciences. Sci. Total Environ..

[94] Association A.P. (2013). Diagnostic and Statistical Manual of Mental Disorders.

[95] WHO (2013). Comprehensive Mental Health Action Plan 2013–2020.

[96] Bratman G.N., Anderson C.B., Berman M.G., Cochran B., de Vries S., Flanders J., Folke C., Frumkin H., Gross J.J., Hartig T. (2019). Nature and mental health: An ecosystem service perspective. Sci. Adv..

[97] Tzoulas K., Korpela K., Venn S., Yli-Pelkonen V., Kaźmierczak A., Niemela J., James P. (2007). Promoting ecosystem and human health in urban areas using Green Infrastructure: A literature review. Landsc. Urban Plan..

[98] Arnberger A., Eder R. (2015). Are urban visitors’ general preferences for green-spaces similar to their preferences when seeking stress relief?. Urban For. Urban Green..

[99] Motazedian A., Coutts A.M., Tapper N.J. (2020). The microclimatic interaction of a small urban park in central Melbourne with its surrounding urban environment during heat events. Urban For. Urban Green..

[100] Langellier B.A., Kuhlberg J.A., Ballard E.A., Slesinski S.C., Stankov I., Gouveia N., Meisel J.D., Kroker-Lobos M.F., Sarmiento O.L., Caiaffa W.T. (2019). Using community-based system dynamics modeling to understand the complex systems that influence health in cities: The SALURBAL study. Health Place.

[101] Ye T., Yu P., Wen B., Yang Z., Huang W., Guo Y., Abramson M.J., Li S. (2022). Greenspace and health outcomes in children and adolescents: A systematic review. Environ. Pollut..

[102] McCormick R. (2017). Does Access to Green Space Impact the Mental Well-being of Children: A Systematic Review. J. Pediatr. Nurs..

[103] de Keijzer C., Bauwelinck M., Dadvand P. (2020). Long-Term Exposure to Residential Greenspace and Healthy Ageing: A Systematic Review. Curr. Environ. Health Rep..

[104] Zhang Y., Wu T., Yu H., Fu J., Xu J., Liu L., Tang C., Li Z. (2024). Green spaces exposure and the risk of common psychiatric disorders: A meta-analysis. SSM Popul. Health.

[105] Gascon M., Triguero-Mas M., Martínez D., Dadvand P., Forns J., Plasència A., Nieuwenhuijsen M.J. (2015). Mental health benefits of long-term exposure to residential green and blue spaces: A systematic review. Int. J. Environ. Res. Public Health.

[106] Wang H., Su T., Zhao W. (2025). Understanding Urban Park-Based Social Interaction in Shanghai During the COVID-19 Pandemic: Insights from Large-Scale Social Media Analysis. ISPRS Int. J. Geo-Information.

[107] Wang Y. (2025). Healing through nature: Public engagement with urban green-blue spaces in pandemic-era NW China. Environ. Impact Assess. Rev..

[108] Fuller R.A., Irvine K.N., Devine-Wright P., Warren P.H., Gaston K.J. (2007). Psychological benefits of greenspace increase with biodiversity. Biol. Lett..

[109] Methorst J., Bonn A., Marselle M., Böhning-Gaese K., Rehdanz K. (2021). Species richness is positively related to mental health—A study for Germany. Landsc. Urban Plan..

[110] Tyrväinen L., Ojala A., Korpela K., Lanki T., Tsunetsugu Y., Kagawa T. (2014). The influence of urban green environments on stress relief measures: A field experiment. J. Environ. Psychol..

[111] Yang W., Yang R., Li X. (2023). A Canonical Correlation Analysis Study on the Association Between Neighborhood Green Space and Residents’ Mental Health. J. Urban Health.

[112] Maas J., Verheij R.A., de Vries S., Spreeuwenberg P., Schellevis F.G., Groenewegen P.P. (2009). Morbidity is related to a green living environment. J. Epidemiol. Community Health.

[113] Sugiyama T., Leslie E., Giles-Corti B., Owen N. (2008). Associations of neighbourhood greenness with physical and mental health: Do walking, social coherence and local social interaction explain the relationships?. J. Epidemiol Community Health.

[114] van den Berg A.E., Maas J., Verheij R.A., Groenewegen P.P. (2010). Green space as a buffer between stressful life events and health. Soc. Sci. Med..

[115] González-Marín A., Garrido-Cumbrera M. (2024). Did the COVID-19 pandemic influence access to green spaces? Results of a literature review during the first year of pandemic. Landsc. Ecol..

[116] Malik K., Kim S., Cultice B.J. (2023). The impact of remote work on green space values in regional housing markets. J. Hous. Econ..

[117] Meschini M., Robinson L., Culhane F., Goffredo S. (2024). Exploring a Bottom-Up Approach to Understanding People’s Relationships with Nature and Their Values of Natural Environment.

[118] Sandstrom G.M., Dunn E.W. (2014). Social Interactions and Well-Being: The Surprising Power of Weak Ties. Pers. Soc. Psychol. Bull..

[119] Arda-Ergen Z. (2025). Sustainability and Urban Quality of Life.

[120] Bikomeye J.C., Namin S., Anyanwu C., Rublee C.S., Ferschinger J., Leinbach K., Lindquist P., Hoppe A., Hoffman L., Hegarty J. (2021). Resilience and Equity in a Time of Crises: Investing in Public Urban Greenspace Is Now More Essential Than Ever in the US and Beyond. Int. J. Environ. Res. Public Health.

[121] Wdowicka M., Mierzejewska L., Szejnfeld M., Modrzewski B., Sikorska-Podyma K., Wronkowski A., Lechowska E. (2024). How to Create Healthy, Stress-Resilient Post-Pandemic Cities. Sustainability.

[122] Hartig T., Mitchell R., de Vries S., Frumkin H. (2014). Nature and health. Annu. Rev. Public Health.

[123] Keniger L.E., Gaston K.J., Irvine K.N., Fuller R.A. (2013). What are the benefits of interacting with nature?. Int. J. Environ. Res. Public Health.

[124] Lanki T., Siponen T., Ojala A., Korpela K., Pennanen A., Tiittanen P., Tsunetsugu Y., Kagawa T., Tyrväinen L. (2017). Acute effects of visits to urban green environments on cardiovascular physiology in women: A field experiment. Environ. Res..

[125] Ikei H., Song C., Sagasaki Y., Nozaki H., Miyazaki Y. (2025). Physiological effects of a small green space installed on the side of a clinic for outpatients with depression. Front. Environ. Health.

[126] Aras S.G., Runyon J.R., Kazman J.B., Thayer J.F., Sternberg E.M., Deuster P.A. (2024). Is Greener Better? Quantifying the Impact of a Nature Walk on Stress Reduction Using HRV and Saliva Cortisol Biomarkers. Int. J. Environ. Res. Public Health.

[127] Kabisch N., van den Bosch M., Lafortezza R. (2017). The health benefits of nature-based solutions to urbanization challenges for children and the elderly—A systematic review. Environ. Res..

[128] Aspinall P., Mavros P., Coyne R., Roe J. (2015). The urban brain: Analysing outdoor physical activity with mobile EEG. Br. J. Sports Med..

[129] Tilley S., Neale C., Patuano A., Cinderby S. (2017). Older People’s Experiences of Mobility and Mood in an Urban Environment: A Mixed Methods Approach Using Electroencephalography (EEG) and Interviews. Int. J. Environ. Res. Public Health.

[130] Dickerson S.S., Kemeny M.E. (2004). Acute stressors and cortisol responses: A theoretical integration and synthesis of laboratory research. Psychol. Bull..

[131] Li Q., Kobayashi M., Kumeda S., Ochiai T., Miura T., Kagawa T., Imai M., Wang Z., Otsuka T., Kawada T. (2016). Effects of Forest Bathing on Cardiovascular and Metabolic Parameters in Middle-Aged Males. Evid. Based Complement Altern. Med..

[132] Schwarz N. (1999). Self-reports: How the questions shape the answers. Am. Psychol..

[133] Kondo M.C., Fluehr J.M., McKeon T., Branas C.C. (2018). Urban Green Space and Its Impact on Human Health. Int. J. Environ. Res. Public Health.

[134] Brauer M., Roth G.A., Aravkin A.Y., Zheng P., Abate K.H., Abate Y.H., Abbafati C., Abbasgholizadeh R., Abbasi M.A., Abbasian M. (2024). Global burden and strength of evidence for 88 risk factors in 204 countries and 811 subnational locations, 1990–2021: A systematic analysis for the Global Burden of Disease Study 2021. Lancet.

[135] Williams D.N., Williams K.A. (2020). Sample Size Considerations: Basics for Preparing Clinical or Basic Research. Ann. Nucl. Cardiol..

[136] Ilker E., Sulaiman Abubakar M., Rukayya Sunusi A. (2015). Comparison of Convenience Sampling and Purposive Sampling. Am. J. Theor. Appl. Stat..

[137] Ford I., Norrie J. (2016). Pragmatic Trials. N. Engl. J. Med..

[138] Xian Z., Nakaya T., Liu K., Zhao B., Zhang J., Zhang J., Lin Y., Zhang J. (2024). The effects of neighbourhood green spaces on mental health of disadvantaged groups: A systematic review. Humanit. Soc. Sci. Commun..

[139] Li J., Cai X., Wamsiedel M. (2024). Perceptions, opportunities and barriers of social engagement among the Chinese older adults: A qualitative study. BMC Geriatr..

[140] Min B., Lee J. (2006). Children’s neighborhood place as a psychological and behavioral domain. J. Environ. Psychol..

[141] Jim C., Shan X. (2013). Socioeconomic effect on perception of urban green spaces in Guangzhou, China. Cities.

[142] Nordh H., Østby K. (2013). Pocket parks for people—A study of park design and use. Urban For. Urban Green..

[143] Balseviciene B., Sinkariova L., Grazuleviciene R., Andrusaityte S., Uzdanaviciute I., Dedele A., Nieuwenhuijsen M.J. (2014). Impact of Residential Greenness on Preschool Children’s Emotional and Behavioral Problems. Int. J. Environ. Res. Public Health.

[144] Dzhambov A.M., Dimitrova D.D. (2014). Elderly visitors of an urban park, health anxiety and individual awareness of nature experiences. Urban For. Urban Green..

[145] Flouri E., Midouhas E., Joshi H. (2014). The role of urban neighbourhood green space in children’s emotional and behavioural resilience. J. Environ. Psychol..

[146] Dzhambov A.M., Dimitrova D.D. (2015). Green spaces and environmental noise perception. Urban For. Urban Green..

[147] Song C., Ikei H., Igarashi M., Takagaki M., Miyazaki Y. (2015). Physiological and Psychological Effects of a Walk in Urban Parks in Fall. Int. J. Environ. Res. Public Health.

[148] Thomas F. (2015). The role of natural environments within women’s everyday health and wellbeing in Copenhagen, Denmark. Health Place.

[149] Wilkie S., Clouston L. (2015). Environment preference and environment type congruence: Effects on perceived restoration potential and restoration outcomes. Urban For. Urban Green..

[150] Zhang Y., Van Dijk T., Tang J., van den Berg A.E. (2015). Green Space Attachment and Health: A Comparative Study in Two Urban Neighborhoods. Int. J. Environ. Res. Public Health.

[151] Akpinar A. (2016). Factors influencing the use of urban greenways: A case study of Aydın, Turkey. Urban For. Urban Green..

[152] Grazuleviciene R., Vencloviene J., Kubilius R., Grizas V., Danileviciute A., Dedele A., Andrusaityte S., Vitkauskiene A., Steponaviciute R., Nieuwenhuijsen M.J. (2016). Tracking Restoration of Park and Urban Street Settings in Coronary Artery Disease Patients. Int. J. Environ. Res. Public Health.

[153] Hadavi S., Kaplan R. (2016). Neighborhood satisfaction and use patterns in urban public outdoor spaces: Multidimensionality and two-way relationships. Urban For. Urban Green..

[154] Sang Å.O., Knez I., Gunnarsson B., Hedblom M. (2016). The effects of naturalness, gender, and age on how urban green space is perceived and used. Urban For. Urban Green..

[155] Staats H., Jahncke H., Herzog T.R., Hartig T. (2016). Urban Options for Psychological Restoration: Common Strategies in Everyday Situations. PLoS ONE.

[156] Hadavi S. (2017). Direct and Indirect Effects of the Physical Aspects of the Environment on Mental Well-Being. Environ. Behav..

[157] Mukherjee D., Safraj S., Tayyab M., Shivashankar R., Patel S.A., Narayanan G., Ajay V.S., Ali M.K., Narayan K.V., Tandon N. (2017). Park availability and major depression in individuals with chronic conditions: Is there an association in urban India?. Health Place.

[158] Dzhambov A., Hartig T., Markevych I., Tilov B., Dimitrova D. (2018). Urban residential greenspace and mental health in youth: Different approaches to testing multiple pathways yield different conclusions. Environ. Res..

[159] Dzhambov A.M., Markevych I., Hartig T., Tilov B., Arabadzhiev Z., Stoyanov D., Gatseva P., Dimitrova D.D. (2018). Multiple pathways link urban green- and bluespace to mental health in young adults. Environ. Res..

[160] Koohsari M.J., Badland H., Mavoa S., Villanueva K., Francis J., Hooper P., Owen N., Giles-Corti B. (2018). Are public open space attributes associated with walking and depression?. Cities.

[161] McEachan R.R.C., Yang T.C., Roberts H., Pickett K.E., Arseneau-Powell D., Gidlow C.J., Wright J., Nieuwenhuijsen M. (2018). Availability, use of, and satisfaction with green space, and children’s mental wellbeing at age 4 years in a multicultural, deprived, urban area: Results from the Born in Bradford cohort study. Lancet Planet. Health.

[162] Nath T.K., Zhe Han S.S., Lechner A.M. (2018). Urban green space and well-being in Kuala Lumpur, Malaysia. Urban For. Urban Green..

[163] Pazhouhanfar M. (2018). Role of Space Qualities of Urban Parks on Mood Change. Psychol. Stud..

[164] Roberts H., Resch B., Sadler J., Chapman L., Petutschnig A., Zimmer S. (2018). Investigating the Emotional Responses of Individuals to Urban Green Space Using Twitter Data: A Critical Comparison of Three Different Methods of Sentiment Analysis. Urban Plan..

[165] Swierad E.M., Huang T.T.K. (2018). An Exploration of Psychosocial Pathways of Parks’ Effects on Health: A Qualitative Study. Int. J. Environ. Res. Public Health.

[166] Tabrizian P., Baran P.K., Smith W.R., Meentemeyer R.K. (2018). Exploring perceived restoration potential of urban green enclosure through immersive virtual environments. J. Environ. Psychol..

[167] Tesler R., Plaut P., Endvelt R. (2018). The Effects of an Urban Forest Health Intervention Program on Physical Activity, Substance Abuse, Psychosomatic Symptoms, and Life Satisfaction among Adolescents. Int. J. Environ. Res. Public Health.

[168] An B.-Y., Wang D., Liu X.-J., Guan H.-M., Wei H.-X., Ren Z.-B. (2018). The effect of environmental factors in urban forests on blood pressure and heart rate in university students. J. For. Res..

[169] Chiang Y.-C., Li D. (2019). Metric or topological proximity? The associations among proximity to parks, the frequency of residents’ visits to parks, and perceived stress. Urban For. Urban Green..

[170] Elsadek M., Liu B., Lian Z., Xie J. (2019). The influence of urban roadside trees and their physical environment on stress relief measures: A field experiment in Shanghai. Urban For. Urban Green..

[171] Macintyre V.G., Cotterill S., Anderson J., Phillipson C., Benton J.S., French D.P. (2019). “I Would Never Come Here Because I’ve Got My Own Garden”: Older Adults’ Perceptions of Small Urban Green Spaces. Int. J. Environ. Res. Public Health.

[172] Schnell I., Harel N., Mishori D. (2019). The benefits of discrete visits in urban parks. Urban For. Urban Green..

[173] Schwartz A.J., Dodds P.S., O’Neil-Dunne J.P.M., Danforth C.M., Ricketts T.H. (2019). Visitors to urban greenspace have higher sentiment and lower negativity on Twitter. People Nat..

[174] Sefcik J.S., Kondo M.C., Klusaritz H., Sarantschin E., Solomon S., Roepke A., South E.C., Jacoby S.F. (2019). Perceptions of Nature and Access to Green Space in Four Urban Neighborhoods. Int. J. Environ. Res. Public Health.

[175] Vujcic M., Tomicevic-Dubljevic J., Zivojinovic I., Toskovic O. (2019). Connection between urban green areas and visitors’ physical and mental well-being. Urban For. Urban Green..

[176] Wang R., Helbich M., Yao Y., Zhang J., Liu P., Yuan Y., Liu Y. (2019). Urban greenery and mental wellbeing in adults: Cross-sectional mediation analyses on multiple pathways across different greenery measures. Environ. Res..

[177] Zhang L., Tan P.Y. (2019). Associations between Urban Green Spaces and Health are Dependent on the Analytical Scale and How Urban Green Spaces are Measured. Int. J. Environ. Res. Public Health.

[178] Zhang T., Liu J., Li H. (2019). Restorative Effects of Multi-Sensory Perception in Urban Green Space: A Case Study of Urban Park in Guangzhou, China. Int. J. Environ. Res. Public Health.

[179] Birch J., Rishbeth C., Payne S.R. (2020). Nature doesn’t judge you—How urban nature supports young people’s mental health and wellbeing in a diverse UK city. Health Place.

[180] Braçe O., Garrido-Cumbrera M., Foley R., Correa-Fernández J., Suárez-Cáceres G., Lafortezza R. (2020). Is a View of Green Spaces from Home Associated with a Lower Risk of Anxiety and Depression?. Int. J. Environ. Res. Public Health.

[181] Campagnaro T., Vecchiato D., Arnberger A., Celegato R., Da Re R., Rizzetto R., Semenzato P., Sitzia T., Tempesta T., Cattaneo D. (2020). General, stress relief and perceived safety preferences for green spaces in the historic city of Padua (Italy). Urban For. Urban Green..

[182] Elsadek M., Liu B., Xie J. (2020). Window view and relaxation: Viewing green space from a high-rise estate improves urban dwellers’ wellbeing. Urban For. Urban Green..

[183] Gagliardi C., Pillemer K., Gambella E., Piccinini F., Fabbietti P. (2020). Benefits for Older People Engaged in Environmental Volunteering and Socializing Activities in City Parks: Preliminary Results of a Program in Italy. Int. J. Environ. Res. Public Health.

[184] Jahani A., Saffariha M. (2020). Aesthetic preference and mental restoration prediction in urban parks: An application of environmental modeling approach. Urban For. Urban Green..

[185] Lopes S., Lima M., Silva K. (2020). Nature can get it out of your mind: The rumination reducing effects of contact with nature and the mediating role of awe and mood. J. Environ. Psychol..

[186] Marselle M.R., Bowler D.E., Watzema J., Eichenberg D., Kirsten T., Bonn A. (2020). Urban street tree biodiversity and antidepressant prescriptions. Sci. Rep..

[187] Mears M., Brindley P., Jorgensen A., Maheswaran R. (2020). Population-level linkages between urban greenspace and health inequality: The case for using multiple indicators of neighbourhood greenspace. Health Place.

[188] Olszewska-Guizzo A., Sia A., Fogel A., Ho R. (2020). Can Exposure to Certain Urban Green Spaces Trigger Frontal Alpha Asymmetry in the Brain?—Preliminary Findings from a Passive Task EEG Study. Int. J. Environ. Res. Public Health.

[189] Saadi D., Schnell I., Tirosh E., Basagaña X., Agay-Shay K. (2020). There’s no place like home? The psychological, physiological, and cognitive effects of short visits to outdoor urban environments compared to staying in the indoor home environment, a field experiment on women from two ethnic groups. Environ. Res..

[190] Tao Y., Yang J., Chai Y. (2019). The Anatomy of Health-Supportive Neighborhoods: A Multilevel Analysis of Built Environment, Perceived Disorder, Social Interaction and Mental Health in Beijing. Int. J. Environ. Res. Public Health.

[191] Aliyas Z. (2019). Physical, mental, and physiological health benefits of green and blue outdoor spaces among elderly people. Int. J. Environ. Health Res..

[192] Bazrafshan M., Tabrizi A.M., Bauer N., Kienast F. (2021). Place attachment through interaction with urban parks: A cross-cultural study. Urban For. Urban Green..

[193] Chu Y.-T., Li D., Chang P.-J. (2021). Effects of Urban Park Quality, Environmental Perception, and Leisure Activity on Well-Being among the Older Population. Int. J. Environ. Res. Public Health.

[194] Du H., Zhou F., Cai Y., Li C., Xu Y. (2021). Research on public health and well-being associated to the vegetation configuration of urban green space, a case study of Shanghai, China. Urban For. Urban Green..

[195] Erdönmez C., Atmiş E. (2021). The impact of the Covid-19 pandemic on green space use in Turkey: Is closing green spaces for use a solution?. Urban For. Urban Green..

[196] Fisher J.C., Bicknell J.E., Irvine K.N., Hayes W.M., Fernandes D., Mistry J., Davies Z.G. (2021). Bird diversity and psychological wellbeing: A comparison of green and coastal blue space in a neotropical city. Sci. Total Environ..

[197] Fisher J.C., Irvine K.N., Bicknell J.E., Hayes W.M., Fernandes D., Mistry J., Davies Z.G. (2021). Perceived biodiversity, sound, naturalness and safety enhance the restorative quality and wellbeing benefits of green and blue space in a neotropical city. Sci. Total Environ..

[198] Høj S.B., Paquet C., Caron J., Daniel M. (2021). Relative ‘greenness’ and not availability of public open space buffers stressful life events and longitudinal trajectories of psychological distress. Health Place.

[199] Mayen Huerta C., Cafagna G. (2021). Snapshot of the Use of Urban Green Spaces in Mexico City during the COVID-19 Pandemic: A Qualitative Study. Int. J. Environ. Res. Public Health.

[200] Jakstis K., Fischer L.K. (2021). Urban Nature and Public Health: How Nature Exposure and Sociocultural Background Relate to Depression Risk. Int. J. Environ. Res. Public Health.

[201] Jing F., Liu L., Zhou S., Song J., Wang L., Zhou H., Wang Y., Ma R. (2021). Assessing the Impact of Street-View Greenery on Fear of Neighborhood Crime in Guangzhou, China. Int. J. Environ. Res. Public Health.

[202] Khalilnezhad M.R., Ugolini F., Massetti L. (2021). Attitudes and Behaviors toward the Use of Public and Private Green Space during the COVID-19 Pandemic in Iran. Land.

[203] Lauwers L., Leone M., Guyot M., Pelgrims I., Remmen R., Broeck K.V.D., Keune H., Bastiaens H. (2021). Exploring how the urban neighborhood environment influences mental well-being using walking interviews. Health Place.

[204] Li H., Liu H., Yang Z., Bi S., Cao Y., Zhang G. (2020). The Effects of Green and Urban Walking in Different Time Frames on Physio-Psychological Responses of Middle-Aged and Older People in Chengdu, China. Int. J. Environ. Res. Public Health.

[205] Liu Q., Zhu Z., Zhuo Z., Huang S., Zhang C., Shen X., Bosch C.C.K.v.D., Huang Q., Lan S. (2021). Relationships between residents’ ratings of place attachment and the restorative potential of natural and urban park settings. Urban For. Urban Green..

[206] Maurer M., Zaval L., Orlove B., Moraga V., Culligan P. (2021). More than nature: Linkages between well-being and greenspace influenced by a combination of elements of nature and non-nature in a New York City urban park. Urban For. Urban Green..

[207] Mawani F.N., Ibrahim S. (2020). Building Roads Together: A peer-led, community-based walking and rolling peer support program for inclusion and mental health. Can. J. Public Health.

[208] McEwan K., Richardson M., Sheffield D., Ferguson F.J., Brindley P. (2021). Assessing the feasibility of public engagement in a smartphone app to improve well-being through nature connection (Evaluación de la factibilidad de la implicación ciudadana mediante una app de teléfonos inteligentes para mejorar el bienestar a través de la conexión con la naturaleza). PsyEcology.

[209] Misiune I., Julian J.P., Veteikis D. (2021). Pull and push factors for use of urban green spaces and priorities for their ecosystem services: Case study of Vilnius, Lithuania. Urban For. Urban Green..

[210] Mohamad N.A., Hussein H. (2020). Perceived Effect of Urban Park as a Restorative Environment for Well Being in Kuala Lumpur. Int. J. Built Environ. Sustain..

[211] Nghiem T., Wong K., Jeevanandam L., Chang C., Tan L., Goh Y., Carrasco L. (2021). Biodiverse urban forests, happy people: Experimental evidence linking perceived biodiversity, restoration, and emotional wellbeing. Urban For. Urban Green..

[212] Olszewska-Guizzo A., Mukoyama A., Naganawa S., Dan I., Husain S.F., Ho C.S., Ho R. (2021). Hemodynamic Response to Three Types of Urban Spaces before and after Lockdown during the COVID-19 Pandemic. Int. J. Environ. Res. Public Health.

[213] Oswald T.K., Rumbold A.R., Kedzior S.G.E., Kohler M., Moore V.M. (2021). Mental Health of Young Australians during the COVID-19 Pandemic: Exploring the Roles of Employment Precarity, Screen Time, and Contact with Nature. Int. J. Environ. Res. Public Health.

[214] Pérez-Del-Pulgar C., Anguelovski I., Cole H.V., de Bont J., Connolly J., Baró F., Díaz Y., Fontán-Vela M., Duarte-Salles T., Triguero-Mas M. (2021). The relationship between residential proximity to outdoor play spaces and children’s mental and behavioral health: The importance of neighborhood socio-economic characteristics. Environ. Res..

[215] Qiao Y., Chen Z., Chen Y., Zheng T. (2021). Deciphering the Link Between Mental Health and Green Space in Shenzhen, China: The Mediating Impact of Residents’ Satisfaction. Front. Public Health.

[216] Ríos-Rodríguez M.L., Rosales C., Lorenzo M., Muinos G., Hernández B. (2021). Influence of Perceived Environmental Quality on the Perceived Restorativeness of Public Spaces. Front. Psychol..

[217] Roberts M., Irvine K.N., McVittie A. (2021). Associations between greenspace and mental health prescription rates in urban areas. Urban For. Urban Green..

[218] Wang X., Zhou Q., Zhang M., Zhang Q. (2021). Exercise in the Park or Gym? The Physiological and Mental Responses of Obese People Walking in Different Settings at Different Speeds: A Parallel Group Randomized Trial. Front. Psychol..

[219] Wang Z., Miao Y., Xu M., Zhu Z., Qureshi S., Chang Q. (2021). Revealing the differences of urban parks’ services to human wellbeing based upon social media data. Urban For. Urban Green..

[220] Wei H., Hauer R.J., Guo S. (2021). Daytime dynamic of spontaneous expressions of pedestrians in an urban forest park. Urban For. Urban Green..

[221] Wu Y., Zhuo Z., Liu Q., Yu K., Huang Q., Liu J. (2021). The Relationships between Perceived Design Intensity, Preference, Restorativeness and Eye Movements in Designed Urban Green Space. Int. J. Environ. Res. Public Health.

[222] Xu J., Wang F., Chen L., Zhang W. (2021). Perceived urban green and residents’ health in Beijing. SSM-Popul. Health.

[223] Zayas-Costa M., Cole H.V.S., Anguelovski I., Connolly J.J.T., Bartoll X., Triguero-Mas M. (2021). Mental Health Outcomes in Barcelona: The Interplay between Gentrification and Greenspace. Int. J. Environ. Res. Public Health.

[224] Zhang L., Liu S., Liu S. (2021). Mechanisms Underlying the Effects of Landscape Features of Urban Community Parks on Health-Related Feelings of Users. Int. J. Environ. Res. Public Health.

[225] Zhang L., Zhou S., Kwan M.-P., Shen M. (2021). Assessing individual environmental exposure derived from the spatiotemporal behavior context and its impacts on mental health. Health Place.

[226] Addas A., Maghrabi A. (2022). How did the COVID-19 pandemic impact urban green spaces? A multi-scale assessment of Jeddah megacity (Saudi Arabia). Urban For. Urban Green..

[227] Ali J., Rahaman M., Hossain S.I. (2022). Urban green spaces for elderly human health: A planning model for healthy city living. Land Use Policy.

[228] Bao Y., Gao M., Luo D., Zhou X. (2022). The influence of outdoor play spaces in urban parks on children’s social anxiety. Front. Public Health.

[229] Chen L., Liu L., Wu H., Peng Z., Sun Z. (2022). Change of Residents’ Attitudes and Behaviors toward Urban Green Space Pre- and Post- COVID-19 Pandemic. Land.

[230] Cheung S.Y.S., Lei D., Chan F.Y.F., Tieben H. (2022). Public Space Usage and Well-Being: Participatory Action Research with Vulnerable Groups in Hyper-Dense Environments. Urban Plan..

[231] Choi K.-A., Rezaei M. (2022). Assessing the Correlation between Neighborhood Green Areas and the Perceived Mental Health of Residents in Metropolitan Areas. Iran. J. Public Health.

[232] Collins C., Haase D., Heiland S., Kabisch N. (2022). Urban green space interaction and wellbeing—Investigating the experience of international students in Berlin during the first COVID-19 lockdown. Urban For. Urban Green..

[233] Flowers E.P., Turner A.I., Abbott G., Timperio A., Salmon J., Veitch J. (2022). People with the least positive attitudes to green exercise derive most anxiolytic benefit from walking in green space. Urban For. Urban Green..

[234] Jin Z., Wang J., Liu X. (2022). Attention and Emotion Recovery Effects of Urban Parks during COVID-19—Psychological Symptoms as Moderators. Forests.

[235] Lee S., Song T., Lim U. (2022). How are happy and unhappy people differently affected by their local environments? The heterogeneous relationship between happiness and local environments in Seoul, Korea. Cities.

[236] Liang H., Yan Q., Yan Y., Zhang L., Zhang Q. (2022). Spatiotemporal Study of Park Sentiments at Metropolitan Scale Using Multiple Social Media Data. Land.

[237] Liu L., Qu H., Ma Y., Wang K., Qu H. (2022). Restorative benefits of urban green space: Physiological, psychological restoration and eye movement analysis. J. Environ. Manag..

[238] Macaulay R., Lee K., Johnson K., Williams K. (2022). ‘Letting my mind run wild’: Exploring the role of individual engagement in nature experiences. Urban For. Urban Green..

[239] MacKinnon M., MacKinnon R., Zari M.P., Glensor K., Park T. (2022). Urgent Biophilia: Green Space Visits in Wellington, New Zealand, during the COVID-19 Lockdowns. Land.

[240] Marchi V., Speak A., Ugolini F., Sanesi G., Carrus G., Salbitano F. (2022). Attitudes towards urban green during the COVID-19 pandemic via Twitter. Cities.

[241] Marconi P.L., Perelman P.E., Salgado V.G. (2022). Green in times of COVID-19: Urban green space relevance during the COVID-19 pandemic in Buenos Aires City. Urban Ecosyst..

[242] Maury-Mora M., Gómez-Villarino M.T., Varela-Martínez C. (2022). Urban green spaces and stress during COVID-19 lockdown: A case study for the city of Madrid. Urban For. Urban Green..

[243] Noszczyk T., Gorzelany J., Kukulska-Kozieł A., Hernik J. (2021). The impact of the COVID-19 pandemic on the importance of urban green spaces to the public. Land Use Policy.

[244] Rapuano M., Ruotolo F., Ruggiero G., Masullo M., Maffei L., Galderisi A., Palmieri A., Iachini T. (2022). Spaces for relaxing, spaces for recharging: How parks affect people’s emotions. J. Environ. Psychol..

[245] Reid C.E., Rieves E.S., Carlson K. (2022). Perceptions of green space usage, abundance, and quality of green space were associated with better mental health during the COVID-19 pandemic among residents of Denver. PLoS ONE.

[246] Schnell I., Hijazi B., Saadi D., Tirosh E. (2022). Women Emotional, Cognitive and Physiological Modes of Coping with Daily Urban Environments: A Pilot Study. Int. J. Environ. Res. Public Health.

[247] Schwartz A.J., Dodds P.S., O’neil-Dunne J.P.M., Ricketts T.H., Danforth C.M. (2022). Gauging the happiness benefit of US urban parks through Twitter. PLoS ONE.

[248] Shan W., Xiu C., Meng Y. (2022). How to Design Greenway on Urban Land Utilization: Linking Place Preference, Perceived Health Benefit, and Environmental Perception. Int. J. Environ. Res. Public Health.

[249] Sun P., Song Y., Lu W. (2022). Effect of Urban Green Space in the Hilly Environment on Physical Activity and Health Outcomes: Mediation Analysis on Multiple Greenery Measures. Land.

[250] Toselli S., Bragonzoni L., Dallolio L., Grigoletto A., Masini A., Marini S., Barone G., Pinelli E., Zinno R., Mauro M. (2022). The Effects of Park Based Interventions on Health: The Italian Project “Moving Parks”. Int. J. Environ. Res. Public Health.

[251] Veitch J., Timperio A., Salmon J., Hall S.J., Abbott G., Flowers E.P., Turner A.I. (2022). Examination of the acute heart rate and salivary cortisol response to a single bout of walking in urban and green environments: A pilot study. Urban For. Urban Green..

[252] Wang P., Han L., Hao R., Mei R. (2022). Understanding the relationship between small urban parks and mental health: A case study in Shanghai, China. Urban For. Urban Green..

[253] Yoo E.-H., Roberts J.E., Eum Y., Li X., Konty K. (2022). Exposure to urban green space may both promote and harm mental health in socially vulnerable neighborhoods: A neighborhood-scale analysis in New York City. Environ. Res..

[254] Yue Y., Yang D., Van Dyck D. (2022). Urban greenspace and mental health in Chinese older adults: Associations across different greenspace measures and mediating effects of environmental perceptions. Health Place.

[255] Zhang Y., Wang M., Li J., Chang J., Lu H. (2022). Do Greener Urban Streets Provide Better Emotional Experiences? An Experimental Study on Chinese Tourists. Int. J. Environ. Res. Public Health.

[256] Amegah A.K., Yeboah K., Owusu V., Afriyie L., Kyere-Gyeabour E., Appiah D.C., Osei-Kufuor P., Annim S.K., Agyei-Mensah S., Mudu P. (2023). Socio-demographic and neighbourhood factors influencing urban green space use and development at home: A population-based survey in Accra, Ghana. PLoS ONE.

[257] Bao Y., Gao M., Zhao C., Zhou X. (2023). White Spaces Unveiled: Investigating the Restorative Potential of Environmentally Perceived Characteristics in Urban Parks during Winter. Forests.

[258] Barron S., Rugel E.J. (2022). Tolerant greenspaces: Designing urban nature-based solutions that foster social ties and support mental health among young adults. Environ. Sci. Policy.

[259] Cao L., Sun Y., Beckmann-Wübbelt A., Saha S. (2023). Characteristics of urban park recreation and health during early COVID-19 by on-site survey in Beijing. npj Urban Sustain..

[260] Cao S., Shang Z., Li X., Luo H., Sun L., Jiang M., Du J., Fu E., Ma J., Li N. (2023). Cloudy or sunny? Effects of different environmental types of urban green spaces on public physiological and psychological health under two weather conditions. Front. Public Health.

[261] Cao Z., Cao Y., Wu Z. (2023). Associations between greenspace characteristics and population emotion perceptions in three dimensions. Front. Environ. Sci..

[262] Chen M., Lin G. (2023). How perceived sensory dimensions of urban green spaces affect cultural ecosystem benefits: A study on Haizhu Wetland Park, China. Urban For. Urban Green..

[263] Chen S., Sleipness O., Christensen K., Yang B., Wang H. (2023). Developing and testing a protocol to systematically assess social interaction with urban outdoor environment. J. Environ. Psychol..

[264] Fancello G., Vallée J., Sueur C., van Lenthe F.J., Kestens Y., Montanari A., Chaix B. (2023). Micro urban spaces and mental well-being: Measuring the exposure to urban landscapes along daily mobility paths and their effects on momentary depressive symptomatology among older population. Environ. Int..

[265] Guo S., Luo Y., Cao Y., Zhang Y., Yu J. (2023). Cultural ecosystem services show superiority in promoting subjective mental health of senior residents: Evidences from old urban areas of Beijing. Urban For. Urban Green..

[266] Honey-Rosés J., Zapata O. (2023). Green Spaces with Fewer People Improve Self-Reported Affective Experience and Mood. Int. J. Environ. Res. Public Health.

[267] Huang Y., Lin X., Lin S., Chen Z., Fu W., Wang M., Dong J. (2023). Pocket Parks: A New Approach to Improving the Psychological and Physical Health of Recreationists. Forests.

[268] Huerta C.M. (2023). Understanding the pathways between the use of urban green spaces and self-rated health: A case study in Mexico City. PLoS ONE.

[269] Jamalishahni T., Turrell G., Foster S., Davern M., Villanueva K. (2023). Neighbourhood socio-economic disadvantage and loneliness: The contribution of green space quantity and quality. BMC Public Health.

[270] Jeong W., Kang H., Shin S., Patel A., Prachand N., Singh M., Stewart W. (2023). Not all ‘greenness’ is equal: Influence of perceived neighborhood environments on psychological well-being in Chicago. Urban For. Urban Green..

[271] Jin Z., Wang J., Liu X., Han X., Qi J., Wang J. (2022). Stress Recovery Effects of Viewing Simulated Urban Parks: Landscape Types, Depressive Symptoms, and Gender Differences. Land.

[272] King H.-J., Lee H.-Y. (2022). Workplace greenspace exposure and the change in dimensions of mood states: An experimental study in Taiwan. Int. J. Environ. Health Res..

[273] Kodali H.P., Ferris E.B., Wyka K., Evenson K.R., Dorn J.M., Thorpe L.E., Huang T.T.-K. (2023). The association of park use and park perception with quality of life using structural equation modeling. Front. Public Health.

[274] Lak A., Khodakarim S., Myint P.K., Baradaran H.R. (2023). The influencing factors of elder-friendly public open spaces promoting older adults’ health in deprived urban neighborhoods: Partial Least Square Structural Equation Modeling approach. Front. Public Health.

[275] Li J., Huang Z., Zheng D., Zhao Y., Huang P., Huang S., Fang W., Fu W., Zhu Z. (2023). Effect of Landscape Elements on Public Psychology in Urban Park Waterfront Green Space: A Quantitative Study by Semantic Segmentation. Forests.

[276] Li X., Xie D., Zhang X., Hou G. (2023). Study on the influence of residents’ well-being on the use of urban parks and emotional recovery under air pollution environment. Int. J. Environ. Pollut..

[277] Li X., Zhang X., Jia T. (2022). Humanization of nature: Testing the influences of urban park characteristics and psychological factors on collegers’ perceived restoration. Urban For. Urban Green..

[278] Li Y., Zhang J., Jiang B., Li H., Zhao B. (2023). Do All Types of Restorative Environments in the Urban Park Provide the Same Level of Benefits for Young Adults? A Field Experiment in Nanjing, China. Forests.

[279] Lin D., Sun Y., Yang Y., Han Y., Xu C. (2022). Urban park use and self-reported physical, mental, and social health during the COVID-19 pandemic: An on-site survey in Beijing, China. Urban For. Urban Green..

[280] Navarrete-Hernandez P., Laffan K. (2023). The impact of small-scale green infrastructure on the affective wellbeing associated with urban sites. Sci. Rep..

[281] Neale C., Boukhechba M., Cinderby S. (2023). Understanding psychophysiological responses to walking in urban settings in Asia and Africa. J. Environ. Psychol..

[282] Ryan S.C., Sugg M.M., Runkle J.D. (2023). Association between urban greenspace, tree canopy cover and intentional deaths: An exploratory geospatial analysis. Urban For. Urban Green..

[283] Setiowati R., Koestoer R., Mizuno K., Hasibuan H. (2023). Urban green space during the Coronavirus disease pandemic with regard to the socioeconomic characteristics. Glob. J. Environ. Sci. Manag. GJESM.

[284] Simović I., Dubljević J.T., Tošković O., Trkulja M.V., Živojinović I. (2023). Underlying Mechanisms of Urban Green Areas’ Influence on Residents’ Health—A Case Study from Belgrade, Serbia. Forests.

[285] Sun Y., Li F., He T., Meng Y., Yin J., Yim I.S., Xu L., Wu J. (2022). Physiological and affective responses to green space virtual reality among pregnant women. Environ. Res..

[286] Villanueva C.P., Labao R.B.J., Tran K.R.A.G., Gonzalez N.R.B., Luna J.M., Ochava K.M.R., Capio C.M. (2023). Resilience and green spaces: Association with stress among contact centre workers in the Philippines. Health Promot. J. Aust..

[287] Wang Y., Chang Q. (2023). The role of urban parks in affecting health outcomes and the differences between vulnerable groups: Evidence from the central city of Beijing. Urban For. Urban Green..

[288] Wu H., Zhu L., Li J., Zhang N., Sun Y., Tang Y., Wang X., Cheng C. (2023). Evaluation and Optimization of Restorative Environmental Perception of Treetop Trails: The Case of the Mountains-to-Sea Trail, Xiamen, China. Land.

[289] Wu L., Luo S., Li D., Chen Q., Li J., Wen J. (2023). Effects of Deciduous Forests on Adolescent Emotional Health in Urban Areas: An Example from the Autumn Ginkgo Forest in Chengdu. Forests.

[290] Xie X., Li Y., Wang R., Gou Z. (2023). Park Recreation Intention and Satisfaction of Blue-Collar Workers Based on the ACSI Model: A Case Study of Anning Industrial Park in Yunnan. Land.

[291] Yan T., Leng H., Yuan Q. (2023). The Role of “Nostalgia” in Environmental Restorative Effects from the Perspective of Healthy Aging: Taking Changchun Parks as an Example. Land.

[292] Yañez D.V., Barboza E.P., Cirach M., Daher C., Nieuwenhuijsen M., Mueller N. (2023). An urban green space intervention with benefits for mental health: A health impact assessment of the Barcelona “Eixos Verds” Plan. Environ. Int..

[293] Yang Z., Zhao X., Zhu L., Xia Y., Ma Y., Wu J., Xiong X., Yang N., Lu M. (2023). Research on the Healing Potential of Urban Parks from the Perspective of Audio-Visual Integration: A Case Study of Five Urban Parks in Chengdu. Land.

[294] Yeon P.-S., Kim I.-O., Kang S.-N., Lee N.-E., Kim G.-Y., Min G.-M., Chung C.-Y., Lee J.-S., Kim J.-G., Shin W.-S. (2022). Effects of Urban Forest Therapy Program on Depression Patients. Int. J. Environ. Res. Public Health.

[295] Yin S., Chen W.Y., Liu C. (2023). Urban forests as a strategy for transforming towards healthy cities. Urban For. Urban Green..

[296] Zhang G., Wu G., Yang J. (2022). The restorative effects of short-term exposure to nature in immersive virtual environments (IVEs) as evidenced by participants’ brain activities. J. Environ. Manag..

[297] Zhao W., Li X., Zhu X., Ye H., Xu H. (2023). Restorative properties of green sheltered spaces and their morphological characteristics in urban parks. Urban For. Urban Green..

[298] Zhou C., Zhang S., Zhao M., Wang L., Chen J., Liu B. (2023). Investigating the dynamicity of sentiment predictors in urban green spaces: A machine learning-based approach. Urban For. Urban Green..

[299] Zhu X., Zhang Y., Luo Y.Y., Zhao W. (2022). Natural or artificial? Exploring perceived restoration potential of community parks in Winter city. Urban For. Urban Green..

[300] Aguome N.M., Ewurum N.I., Ifeanacho K.P., Abaa-Okorie L.C., Ugwu C.G. (2024). Public recreational facilities as catalyst for urban aging-in-place decision in developing countries. Cities.

[301] Arnberger A., Eder R., Allex B., Wallner P., Weitensfelder L., Hutter H.-P. (2024). Urban green space preferences for various health-related psychological benefits of adolescent pupils, university students and adults. Urban For. Urban Green..

[302] Bock J.L., Nesbitt L., Mavoa S., Meitner M.J. (2024). Attributes and benefits of urban green space visits—Insights from the City of Vancouver. Urban For. Urban Green..

[303] Bressane A., Pinto J.P.d.C., Goulart A.P.G., Medeiros L.C.d.C. (2024). Which dimensions of nature contact in Urban Green Spaces most significantly contribute to mental wellbeing? A multidimensional analysis in Brazilian metropolitan cities. Health Place.

[304] Bressane A., Pinto J.P.d.C., Medeiros L.C.d.C. (2024). Urban green space disparities: Implications of environmental injustice for public health. Urban For. Urban Green..

[305] Chang P.-J., Ho L.-C., Suppakittpaisarn P. (2024). Investigating the interplay between senior-friendly park features, perceived greenness, restorativeness, and well-being in older adults. Urban For. Urban Green..

[306] Chen X., Marzbali M.H., Abdullah A. (2024). Urban Parks and Office Workers’ Health: Considering the Influence of Marital Status and Different Qualities of Urban Parks. Societies.

[307] Chen X., Marzbali M.H. (2024). How urban park features impact perceived safety by considering the role of time spent in the park, gender, and parental status. Cities.

[308] Dong T., Zhong Q., Yue B. (2024). How Green Space Justice in urban built-up areas affects public mental health: A moderated chain mediation model. Front. Public Health.

[309] Felappi J.F., Sommer J.H., Falkenberg T., Terlau W., Kötter T. (2024). Urban park qualities driving visitors mental well-being and wildlife conservation in a Neotropical megacity. Sci. Rep..

[310] Fossa A.J., D’sOuza J., Bergmans R.S., Zivin K., Adar S.D. (2024). Different types of greenspace within urban parks and depressive symptoms among older U.S. adults living in urban areas. Environ. Int..

[311] Han S., Song D., Shi F., Du H., Zhang Y., Yang M. (2024). Assessing Neighbourhood Preference: An Evaluation of Environmental Features within Small-Scale Open Spaces. Land.

[312] Hao J., Li Y., Hu T., Ma Y., Wang X., Liu J., Gao T., Qiu L. (2024). Vegetation diversity in structure, species or colour: Coupling effects of the different characteristics of urban green spaces on preference and perceived restoration. Ecol. Indic..

[313] Hassall C., Nisbet M., Norcliffe E., Wang H. (2024). The Potential Health Benefits of Urban Tree Planting Suggested through Immersive Environments. Land.

[314] Henderson L., Tipper L., Willicombe S., Gattis M. (2024). Shared time in nature increases feelings of social connection amongst university students. J. Environ. Psychol..

[315] Hou J., Wang Y., Zhang X., Qiu L., Gao T. (2024). The effect of visibility on green space recovery, perception and preference. Trees For. People.

[316] Huang J., Song Y., Sheng Y., Zhang Y., Hu D. (2024). Restorative Potential Assessment of Public Open Space in Old Urban Communities in the Context of Aging—A Case Study of Dabeizhuang Community in Maanshan, China. Buildings.

[317] Jin Y., Yu Z., Yang G., Yao X., Hu M., Remme R., van Bodegom P., Morpurgo J., Huang Y., Wang J. (2024). Quantifying physiological health efficiency and benefit threshold of greenspace exposure in typical urban landscapes. Environ. Pollut..

[318] Kruja S., Braçe O., Kokthi E., Cumbrera M.G. (2024). The Role of Urban Green Spaces on Life Satisfaction and Migration Willingness in Tirana, Albania. Hum. Ecol..

[319] Mollaesmaeili M., Hakimian P., Lak A. (2024). Perceived urban green spaces and youth mental health in the post-COVID-19 era. Front. Public Health.

[320] Naghibi M. (2024). Rethinking small vacant lands in urban resilience: Decoding cognitive and emotional responses to cityscapes. Cities.

[321] Nosrati A., Pazhouhanfar M., Chen C., Grahn P. (2024). Designing Stress-Relieving Small Inner-City Park Environments for Teenagers. Land.

[322] Odhengo P., Lutta A.I., Osano P., Opiyo R. (2024). Urban green spaces in rapidly urbanizing cities: A socio-economic valuation of Nairobi City, Kenya. Cities.

[323] Refisch M., Kurz K., Hartmann J. (2024). Urban Green Space Usage and Life Satisfaction During the COVID-19 Pandemic. Appl. Res. Qual. Life.

[324] Ruotolo F., Rapuano M., Masullo M., Maffei L., Ruggiero G., Iachini T. (2023). Well-being and multisensory urban parks at different ages: The role of interoception and audiovisual perception. J. Environ. Psychol..

[325] Sadeghpoor F., Ranjbar E., Esmaeilinasab M., Valiloo M.H.S., Nieuwenhuijsen M.J. (2023). Streets and Stress: A Pilot Study on How Quality and Design of Streets Impacts on Urban Stress. HERD Health Environ. Res. Des. J..

[326] Shahmiri M.S., Boujari P., Dehkordi Z.S.F., Khatami S.M. (2024). Do Green Spaces Mitigate Mental Health Disorders in Tehran? Evidence from 358 Neighborhoods. HERD Health Environ. Res. Des. J..

[327] Shao Y., Yin Y., Ma D. (2023). Evaluating the Equity of Urban Streetscapes in Promoting Human Health—Taking Shanghai Inner City as an Example. Land.

[328] Tohan M.M., Ahmed F., Juie I.J., Kabir A., Rahman A. (2024). Outdoor recreational activities and mental well-being of geriatric people in Bangladesh: Structural equation modelling. Discov. Psychol..

[329] Vegaraju A., Amiri S. (2023). Urban green and blue spaces and general and mental health among older adults in Washington state: Analysis of BRFSS data between 2011–2019. Health Place.

[330] Wan Z., Shen X., Cai Y., Su Y., Ren Z., Xia Y. (2024). How to Make Flower Borders Benefit Public Emotional Health in Urban Green Space: A Perspective of Color Characteristics. Forests.

[331] Wang S., Li A. (2024). Identify the significant landscape characteristics for the perceived restorativeness of 8 perceived sensory dimensions in urban green space. Heliyon.

[332] Wang Y., Shi X., Hong H., Chang Q. (2024). How does multiscale greenspace exposure affect human health? Evidence from urban parks in the central city of Beijing. J. Environ. Manag..

[333] Wang Z., Cheng H., Li Z., Wang G. (2024). Is greener always healthier? Examining the nonlinear relationships between urban green spaces and mental health in Wuhan, China. Urban For. Urban Green..

[334] Wu K., Guo Y., Han X. (2024). The relationship research between restorative perception, local attachment and environmental responsible behavior of urban park recreationists. Heliyon.

[335] Xu J., Qiu B., Zhang F., Zhang J. (2024). Restorative Effects of Pocket Parks on Mental Fatigue among Young Adults: A Comparative Experimental Study of Three Park Types. Forests.

[336] Xu T., Aini A.M., Nordin N.A. (2024). Utilizing regression model to characterize the impact of urban green space features on the subjective well-being of older adults. Heliyon.

[337] Yan T., Leng H., Yuan Z. (2024). Construction of the “Full Path” of restorative effects on older adults’ mental health in parks under seasonal differences: Taking Changchun as an example. Front. Public Health.

[338] Ye X., Chen K., Chen J., Chan I.-T. (2024). Perceptions and Uses of Public Open Spaces During the COVID-19 Pandemic: Prevention Measures as Endangering Possibilities of Active Ageing. SAGE Open.

[339] Ma C.Y., Yang C. (2023). The moderating effects of mobile applications on the use of urban green space and mental health of older people: A mixed-method investigation in Hong Kong. Urban For. Urban Green..

[340] Zhang R., Zhu L., Yang X., Han R., Zhang Y., Kang J. (2024). The effects of interaction with audiovisual elements on perceived restoration in urban parks in freezing weather. Urban For. Urban Green..

[341] Zhang T., Wang L., Zhang Y., Hu Y., Zhang W. (2024). Assessing the nonlinear impact of green space exposure on psychological stress perception using machine learning and street view images. Front. Public Health.

[342] Zhang Y., Zhao J., Mavoa S., Fenaughty J., Clark T.C., Crengle S., Smith M. (2024). Impacts of sociodemographic factors, identities and neighbourhood safety on the relationship between urban green space and adolescent mental well-being: Findings from Tāmaki Makaurau Auckland, Aotearoa New Zealand. SSM-Popul. Health.

[343] Zhang Y., Fang Y., Wang M., Li W. (2024). How Do Plant Landscapes Provide Health Benefits to Residents in Urban Green Spaces? Exploring the Role of Restorative Experiences. Forests.

[344] Zhong S., Ren J. (2024). Evaluation on nature-connected environment in building embedded landscape: Theory, detection, and case design. Front. Environ. Sci..

[345] Zuo W., Cheng B., Feng X., Zhuang X. (2024). Relationship between urban green space and mental health in older adults: Mediating role of relative deprivation, physical activity, and social trust. Front. Public Health.

