# Supplementary material for: Investigation of Epistemic Equity in Urban Green Space and Mental Health Research: A Systematic Review
Source: Int J Environ Res Public Health. 2026 Feb 9;23(2):218. doi: 10.3390/ijerph23020218 (PMC12940324; doi:10.3390/ijerph23020218)
Supplement: Supplementary file 1 [file ijerph-23-00218-s001.zip › Supplementary Figure S1.pdf]

According to the data presented in Figure S1, the number of published studies concerning urban public green spaces and mental health exhibits a marked upward trend between 2006 and 2024. From 2006 to 2016, the volume of related literature remained relatively low, with fewer than 10 publications per year. Beginning in 2017, the number of studies started to rise intermittently, with a pronounced surge in 2020. Specifically, the number of publications increased sharply from 9 in 2019 to 40 in 2021. Although a slight decline was observed in 2022, publication volume rebounded swiftly in 2023, reaching a peak of 49 articles in 2024. This overall trend indicates a substantial increase in scholarly and public interest in the psychological functions of urban green spaces during the COVID-19 pandemic, which significantly accelerated research activity in this domain.

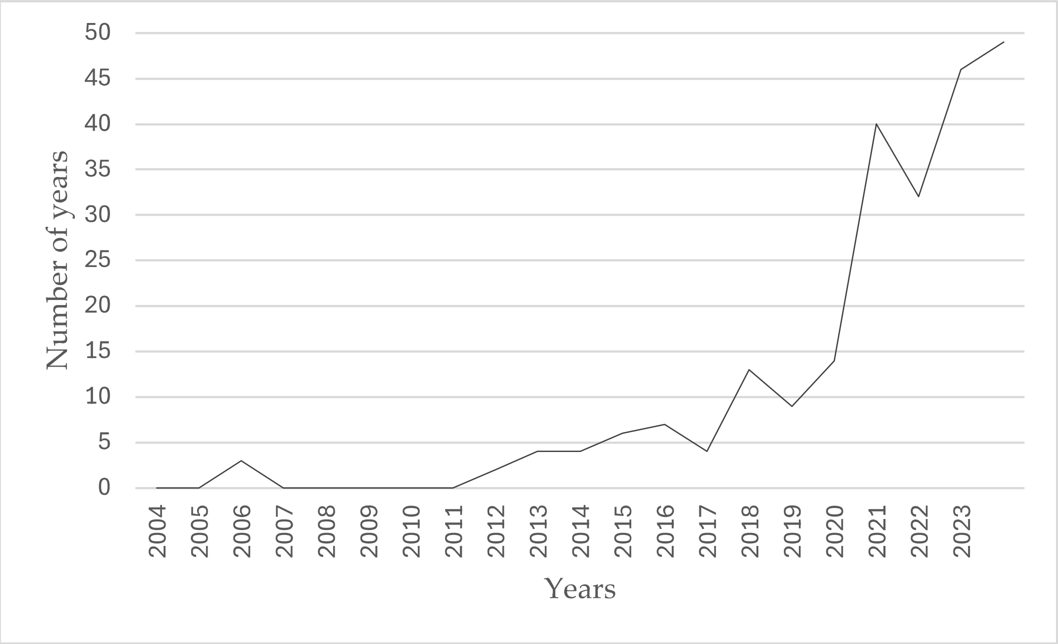

Figure S1. Number of publications per year.
