# Supplementary material for: Investigation of Epistemic Equity in Urban Green Space and Mental Health Research: A Systematic Review
Source: Int J Environ Res Public Health. 2026 Feb 9;23(2):218. doi: 10.3390/ijerph23020218 (PMC12940324; doi:10.3390/ijerph23020218)
Supplement: Supplementary file 1 [file ijerph-23-00218-s001.zip › Supplementary Figure S2.pdf]

Among the five mental health dimensions (Figure S2), the highest concentration of studies is in “Well-Being and Resilience“ encompassing 187 publications and accounting for 79.57% of the total, reflecting strong academic interest in positive psychological states. This is followed by “Emotional Disorders” and “Stress and Trauma” with 133 (56.60%) and 112 (47.66%) studies, respectively, peaking in 2023 and 2024. For instance, 32 studies focusing on emotional disorders were published in 2023, while 38 studies on well-being and resilience appeared in 2024, which represents the highest annual count within the review period. In contrast, the “Sleep and Behavioral Functioning“ and “Neuropsychological and Physiological“ dimensions received comparatively limited attention, with 14 (5.96%) and 32 (13.62%) studies, respectively. Notably, research on “Neuropsychological and Physiological“ outcomes began to increase significantly after 2020, reaching 11 studies in 2023, suggesting that this subfield may represent an emerging area of interest for future scholarly inquiry.

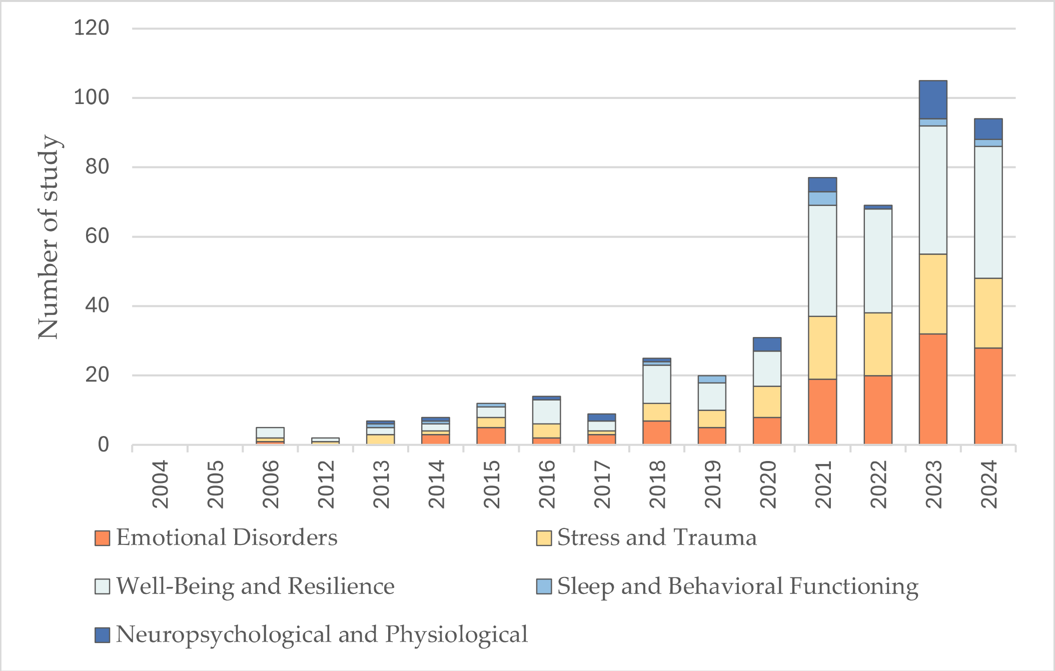

Figure S2. Annual number of studies on mental health dimensions in urban green space and mental health research (2004–2024). The five categorized mental health dimensions are: "Emotional Disorders", "Stress and Trauma", "Well-Being and Resilience", "Sleep and Behavioral Functioning", and "Neuropsychological and Physiological Outcomes".
