# Supplementary material for: Investigation of Epistemic Equity in Urban Green Space and Mental Health Research: A Systematic Review
Source: Int J Environ Res Public Health. 2026 Feb 9;23(2):218. doi: 10.3390/ijerph23020218 (PMC12940324; doi:10.3390/ijerph23020218)
Supplement: Supplementary file 1 [file ijerph-23-00218-s001.zip › Supplementary Table S3.pdf]

In terms of spatial distribution (see Table S3), the studies are concentrated primarily in two regions: Asia and Europe. Asia accounts for the largest share, comprising 55.74% of the total (131 articles), with China contributing the most publications (89 articles). Europe ranks second with 57 studies (24.26%), primarily due to contributions from the United Kingdom (14 articles) and Spain (eight articles). In North America, research is concentrated primarily in the United States, which produced 22 articles, accounting for about 11.91% of the total. In Oceania, most contributions come from Australia with 11 articles (4.68%). In contrast, Africa (three articles, 1.28%) and South America (six articles, 1.7%) exhibit markedly lower research output in this dataset. These patterns indicate a pronounced geographic disparity within the English-language Web of Science literature. These patterns may partly reflect limitations in database coverage and language restrictions and should therefore not be interpreted as evidence that little or no local research has been conducted in underrepresented regions.

Table S3. Continents and countries where studies have been conducted on the mental health role of urban public green spaces. Each study may be conducted in multiple countries, either on the same or different continents.

Continent	Country	Number of Studies (%)	
**Africa**	Kenya	2	0.85%
_Number of studies: 3; 1.28%)	Ghana	1	0.43%
Asia	China	89	37.87%
(Number of studies: 131; 55.74%)	Iran	11	4.68%
	Israel	5	2.13%
	Taiwan	5	2.13%
	Singapore	4	1.70%
	South Korea	4	1.70%
	Malaysia	3	1.28%
	India	2	0.85%
	Bangladesh	1	0.43%
	Hong Kong	1	0.43%
	Indonesia	1	0.43%
	Japan	1	0.43%
	Philippines	1	0.43%
	Saudi Arabia	1	0.43%
	Thailand	1	0.43%
	Turkey	1	0.43%
Europe	United Kingdom	13	5.53%
(Number of studies: 57; 24.26%)	Spain	8	3.40%
	Germany	6	2.55%
	Italy	6	2.55%
	Bulgaria	4	1.70%
	Lithuania	3	1.28%
	Netherlands	3	1.28%
	Finland	2	0.85%
	Serbia	2	0.85%
	Albania	1	0.43%
	Austria	1	0.43%

	Belgium	1	0.43%
	Denmark	1	0.43%
	England	1	0.43%
	France	1	0.43%
	Norway	1	0.43%
	Poland	1	0.43%
	Portugal	1	0.43%
	Sweden	1	0.43%
North America	USA	22	9.36%
(Number of studies: 28; 11.91%)	Canada	3	1.28%
	Mexico	3	1.28%
Oceania	Australia	8	3.40%
(Number of studies: 11; 4.68%)	New Zealand	3	1.28%
South America	Brazil	3	1.28%
(Number of studies: 7; 1.70%)	Guyana	2	0.85%
	Argentina	1	0.43%
	Chile	1	0.43%
