# Supplementary material for: Investigation of Epistemic Equity in Urban Green Space and Mental Health Research: A Systematic Review
Source: Int J Environ Res Public Health. 2026 Feb 9;23(2):218. doi: 10.3390/ijerph23020218 (PMC12940324; doi:10.3390/ijerph23020218)
Supplement: Supplementary file 1 [file ijerph-23-00218-s001.zip › Supplementary Text S1 Retrospective review protocol.pdf]

Supplementary Text S1 Retrospective review protocol

Title: Investigation of Epistemic Equity in Urban Green Space and Mental Health Research: A Systematic Review

Authors: Qin Huang 1, Kun Liu 2, Fupeng Li 3, Yongming Huang 1, Yanggang Huang 4 and Ryosuke Shimoda 1,*

Last Updated: January 18, 2026

1. Rationale and Justification for Non-Registration

This review was conducted in accordance with the PRISMA 2020 guidelines. We acknowledge that the study protocol was not prospectively registered in databases such as PROSPERO. This decision was grounded in the exploratory and theoretical nature of this investigation. Unlike standard systematic reviews aimed at aggregating effect sizes of clinical interventions, this study employs the 'Equity Bias Framework' to map the landscape of 'Epistemic Equity'. This framework specifically integrates the three dimensions of study design, psychometric measures, and population sampling. Given that the Equity Bias Framework required iterative operationalization and refinement during the initial scoping phase, rigid prospective registration was deemed unfeasible. Consequently, we provide this retrospective protocol to ensure methodological transparency and reproducibility.

2. Research Objectives

The primary objective of this review is to evaluate the epistemic equity within the evidence base linking urban green spaces to mental health. Specifically, it addresses three core questions:

(1) Do existing studies exhibit systematic preferences in study design and measures selection?

(2) Are different social groups fairly represented in research samples, or is there a systematic absence of vulnerable or marginalized populations?

(3) How do these biases collectively shape the current evidence structure on urban green spaces and mental health?

3. Eligibility Criteria

Studies were selected based on the following inclusion and exclusion criteria:

3.1 Inclusion Criteria

(1) Timeframe: Studies published between January 1, 2004, and December 31, 2024.

(2) Language: Only publications in English were included.

(3) Document Type: Only peer-reviewed original research articles were included. (Exclusion: Review articles, conference proceedings, editorials, book chapters, and letters were excluded to avoid duplicating secondary evidence).

(4)Exposure（Urban Green Space）: Studies must focus on publicly accessible urban green spaces designed for everyday use by the general public. "Accessibility" is defined by the ability to access the space via walking or public transport within an urban context, and the absence of entry fees or membership requirements. It includes neighborhood, community, and district parks; urban green lots; general-purpose parks; greenways; and street greenery, including stream corridors.

Special Note on Private Gardens: Private or home gardens are excluded if analyzed as private recreational spaces (e.g., gardening activities). However, they are included only if they are analyzed as part of the visual streetscape (e.g., green view visible to pedestrians).

(5) Outcomes (Mental Health): Research must provide empirical validation of the

relationship between urban green spaces and mental health outcomes. This includes: Direct association: Green space characteristics → Mental health outcomes (e.g., depression, anxiety, subjective well-being). Mediated pathways: Green space → Mediating variables (e.g., physical activity, social cohesion) → Mental health outcomes.

(6) Measurement and Data Collection Requirements: Included studies must report explicit methods for assessing mental health or psychological states. For quantitative research, standardized psychometric scales (e.g., PHQ-9, WHO-5), physiological biomarkers (e.g., cortisol), or structured self-report items must be used to evaluate outcomes. For qualitative studies, rigorous qualitative data collection methods (e.g., in-depth interviews, focus groups, participant observation) must be employed to explicitly explore participants' psychological or emotional experiences related to green spaces.

3.2 Exclusion Criteria

(1) Report Characteristics:

①Non-English Publications

②Non-empirical articles: Reviews, conference proceedings, editorials, book chapters, and letters were excluded to avoid duplicating secondary evidence or including non-peer-reviewed data.

(2) Setting and Exposure (Green Space Types):

①Low-Accessibility or Remote Areas: Green spaces with low accessibility, such as national parks, nature reserves, and suburban forest parks, which do not represent everyday urban exposure, or those requiring fees or travel to suburbs.

②Restricted/private spaces: Green spaces intended for exclusive use by specific groups, such as private residential gardens (unless analyzed as streetscape visibility), industrial park greenery, or corporate campuses.

③Commercial or functional spaces: Commercial green spaces, such as golf courses, and functional green infrastructure that lacks public access or a recreational function, such as windbreaks, noise barriers, and ecological conservation zones.

(3) Outcome Focus:

①Ecological Functions Only: Studies that focus solely on ecological metrics (e.g., air pollution removal, soil quality, and biodiversity) without measuring human mental health outcomes. Note: Studies analyzing these ecological factors as mediators linking green space to mental health were included.

②Economic Valuations Only: Studies that focus on the economic impact of green spaces (e.g., real estate values or hedonic pricing). Studies that mentioned mental health benefits in the background or introduction were excluded if they did not empirically measure a psychological outcome.

4. Information Sources and Search Strategy

(1) Database: Web of Science Core Collection.

(2) Search Fields: The search strings were applied to the Topic (TS) field, which searches the Title, Abstract, Author Keywords, and Keywords Plus.

(3) Search String: TS = (("urban" OR "city") AND ("park*" OR "green space" OR "green infrastructure" OR "green corridor*" OR "urban forest") AND ("mental health" OR "depres*" OR "anxi*" OR "stress" OR "psychological" OR "subjective wellbeing" OR "emotional restoration" OR "restorative quality" OR "attention" OR "perceived safety" OR "affect*" OR

"emotion*" OR "mental" OR "percept*" OR "restorative*"))

(3) Filters Applied: Language (English); Document Type (Article); Publication date: between January 2004 and December 2024.

(4) Search Date: April 25, 2025

5. Study Selection Process

5.1 Initial Filtering by Subject Category (WOS):

To ensure feasibility while maintaining interdisciplinary coverage, we applied an initial filter using the Web of Science's "Subject Categories." We retained all categories relevant to the social sciences, psychology, public health, urban planning, and environmental studies.

We excluded categories that were conceptually misaligned with the research question, i.e., those lacking a focus on human mental health or urban green space. These exclusions were grouped into three domains: (1) Non-Human Natural Sciences: Categories focusing on animal biology, atmospheric physics, or geology (e.g., zoology, entomology, meteorology and atmospheric sciences, soil science, and oceanography). (2) Specialized Clinical Medicine and Pathology: Categories focusing on specific organ pathologies, surgery, or cellular mechanisms rather than environmental determinants of health (e.g., anesthesiology, urology, orthopedics, cell biology, parasitology, dentistry, dermatology). (3) Unrelated Technical/Historical Fields: Categories focusing on hardware, logistics, or history (e.g., computer vision, modern history). A manual check of a random sample of excluded records confirmed that studies in these categories primarily investigated biophysical properties, animal models, or clinical surgical techniques with no relevance to the "green space–mental health" nexus. In total, 7,837 unique records were excluded through category-based filtering (see Supplementary Table S1 for the full list of excluded Web of Science topic filters and record counts).

5.2 Screening and Verification Procedure

A multi-stage screening process was employed to ensure reliability and consistency. (1) Criteria Development: One reviewer drafted the initial inclusion/exclusion criteria, which were then discussed and refined with the co-author team (including the corresponding author) to ensure conceptual alignment. (2) Calibration (Pilot Screening): To calibrate the application of these criteria, two reviewers screened a random sample of 80 initial records independently. Each record was marked as "Include," "Exclude," or "Uncertain," and the reasons were recorded. (3) Resolution: Disagreements and "uncertain" cases were discussed with the wider research team. These discussions were used to further refine the decision rules and clarify ambiguous definitions (e.g., the definition of "publicly accessible" green space). (4)Full Screening: After calibration, one reviewer screened the remaining records using the refined criteria. A brief justification was recorded for every decision (e.g., "Excluded — no mental health outcome"). (5) Verification and Consensus: To mitigate single-screener bias, two additional reviewers audited the screening decisions and recorded justifications. Any discrepancies or uncertainties identified were resolved through consensus meetings involving all reviewers until a final decision was reached.

Although a formal Kappa statistic was not calculated for the entire dataset, the structured calibration and audit process acted as a rigorous quality control mechanism, ensuring the validity of the selection process.

For the 235 included studies, one researcher initially extracted and coded key variables (study design, mental health outcome dimensions, green space characteristics, and population

categories) using a standardized extraction template. A second researcher independently verified the accuracy and consistency of all extracted items. Any discrepancies were flagged, discussed, and resolved through consensus with other authors. The final dataset reflects the consensus outcome of three reviewers.
