# Supplementary material for: Investigation of Epistemic Equity in Urban Green Space and Mental Health Research: A Systematic Review
Source: Int J Environ Res Public Health. 2026 Feb 9;23(2):218. doi: 10.3390/ijerph23020218 (PMC12940324; doi:10.3390/ijerph23020218)
Supplement: Supplementary file 1 [file ijerph-23-00218-s001.zip › Supplementary Text S2 Codebook.pdf]

1. Data Extraction and Coding Procedure:

A rigorous, iterative approach was used for the coding process to ensure reliability and consistency across the dataset. The procedure consisted of four distinct stages:

(1) Schema Development: One researcher developed an initial coding schema based on the study objectives and a pilot review of the literature. Key variables and preliminary definitions were established to guide the initial extraction.

(2) Independent coding and verification: One researcher performed the initial coding. To ensure accuracy, a second researcher verified the coded data against the full text of the articles

(3) Iterative Refinement: An inductive approach was adopted during the coding process. When researchers encountered cases that did not fit the initial definitions, such as distinguishing "University Students" from "Healthy Adults" due to unique campus exposures or identifying "Quasi-experimental" designs, these instances were flagged as "Unclassified/Other" and detailed notes were made for review.

(4) Consensus and Retrospective Application: Discrepancies and flagged cases were discussed in consensus meetings with the full research team. New categories were formally defined (e.g., creating a separate category for "University Students"), and the coding schema was updated. Crucially, these refined definitions were applied retroactively to all previously coded records to ensure consistency across the entire dataset.

2. General Coding Rules

Unit of Analysis: Individual study.

Handling of Missing Data: If specific information was not explicitly stated in the text, it was coded as "Not Reported (NR)". Multiple Entries: For studies reporting multiple mental health outcomes or green space types, all applicable categories were recorded to capture the full scope of the research.

For the core dimensions of the Equity Bias Framework (e.g., study design, psychometric measures, and population sampling), coding categories were developed iteratively during pilot extraction so that every included study could be assigned to at least one category. Ambiguous cases were resolved through full-text review and discussion, and no study was coded as "not reported" for these core variables. For secondary descriptive variables (e.g., sample size), instances where information was missing or unclear were coded as "not reported" and excluded from quantitative summaries involving that specific variable. The studies remained in all other analyses.

3. Variable Definitions (Codebook)

The following table 1 details the variables extracted from each included study. Variables marked with an asterisk (*) represent data extracted to provide context or facilitate future research reuse, although they may not have been the primary focus of the manuscript's synthesis.

table 1 details the variables extracted from each included study.

Variable Name	Description / Categories / Coding Rules	Notes / Rationale
**A. Bibliographic and Geographical Information**		

**Article Title**	Full title of the published article.	
**Publication Year**	Year of publication.	
**DOI**	Digital Object Identifier.	
**Continent**	Geographical continent (e.g., Europe, Asia, North America).	
**Country**	Name of the country where the study was conducted.	
**B. Study Design and Methodology**		
**Methodological Approach**	**Quantitative / Qualitative / Mixed Methods**	Broad classification of research methods.
**Study Design**	1. **Cross-sectional study**	**Refined Iteratively:** "Quasi-experimental" and "Case Study" were added to accommodate specific non-randomized or in-depth designs identified during the full-text review.
	2. **Longitudinal study**	
	3. **Experimental research**	
	4. **Quasi-experimental study**	
	5. **Case Study**	
	6. **Qualitative descriptive design**	
**Sample Size (N)** *	Total number of participants included in the analysis.	Data extracted for potential future meta-analysis or reuse.
**population sampling**	1. **Community-dwelling adult populations**: General adults recruited from public areas or workplaces.	**Refined Iteratively:** Distinct subgroups (e.g., **University Students**) were classified under Category 4 due to their unique environmental exposures (campus) and distinct academic stressors compared to general working adults.
	2. **Age-specific vulnerable populations**: Specifically targeting children, adolescents, or elderly groups.	
	3. **Clinical or high-risk populations**: Participants with diagnosed conditions or specific health risks.	
	4. **Institutional or convenience populations**: Specific groups such as university students or prisoners.	

**Reliability Reported ***	**Yes / No**	Indicates if reliability metrics (e.g., Cronbach's alpha) were reported. Extracted for quality assessment context.
**C. Green Space Assessment (Exposure)**		
**Green Space Data Acquisition**	01. **Remote Sensing** (e.g., satellite imagery)	
	02. **Field Survey/Audit** (On-site measurement)	
	03. **Images/Street View** (e.g., Google Street View)	
	04. **VR** (Virtual Reality simulation)	
	05. **Social Media Data**	
	06. **Photography** (Participant/Researcher photos)	
	07. **Other**	
**Green Space Characteristics**	**1. Physical Structure:** Measurable features (e.g., total area, NDVI, canopy cover).	The primary dimensions used for synthesis in the manuscript.
	**2. Ecological Functions:** Regulatory services (e.g., temperature regulation, noise attenuation, biodiversity).	
	**3. Perceived Naturalness and Aesthetics:** Subjective perceptions of visual appeal, spatial enclosure, etc.	
	**4. Social and Recreational Usability:** Accessibility, facilities, and support for social activities.	
**Specific Green Space Type ***	01. **Urban Green Space (General)**	Detailed classification extracted to facilitate future research reuse.
	02. **Urban Parks (General)**	
	03. **Neighborhood Parks**	
	04. **Children’s Parks**	

	05. **Urban Forests**	
	06. **Block Parks**	
	07. **Street Greenery**	
	08. **Other**	
**D. Mental Health Assessment (Outcome)**		
**Mental Health Dimensions**	**(1) Emotional Disorders:** Negative symptoms (Depression, Anxiety; e.g., PHQ-9, GAD-7).	
	**(2) Stress and Trauma:** Psychological stress/PTSD (e.g., PSS).	
	**(3) Well-being and Resilience:** Positive emotions/satisfaction (e.g., WHO-5, WEMWBS).	
	**(4) Sleep and Behavioral Functioning:** Sleep quality, biorhythms, daily efficiency.	
	**(5) Neuropsychological and Physiological:** Cognitive function, HRV, Cortisol, etc.	
**Measurement Method**	1. **Self-report psychometric measures**	
	2. **Clinician-rated psychometric measures**	
	3. **Physiological and biochemical indicators**	
	4. **Behavioral and observational measures**	
**Theoretical Basis ***	01. **Attention Restoration Theory (ART)**	Contextual data extracted to understand the conceptual framework of included studies.
	02. **Stress Recovery Theory (SRT)**	
	03. **Biophilia Hypothesis**	
	04. **Self-Determination Theory (SDT)**	
	05. **Ecosystem Services Theory**	
	06. **Other**	

**E. Analytical Framework**		
**Analytical/Path Type** *	01. **Causal Inference**	Contextual data indicating the primary statistical or qualitative approach used to link green space and mental health.
	02. **Correlation Analysis**	
	03. **Mediation Model**	
	04. **Spatial Regression**	
	05. **Moderation Analysis** (e.g., age as moderator)	
	06. **Thematic Analysis** (Qualitative)	
	07. **Interpretative Phenomenological Analysis (IPA)**	
	08. **Other**	
